# It takes two peroxisome proliferator-activated receptors (PPAR-β/δ and PPAR-γ) to tango idiopathic pulmonary fibrosis

**DOI:** 10.1186/s12931-024-02935-7

**Published:** 2024-09-23

**Authors:** Eistine Boateng, Rocio Bonilla-Martinez, Barbara Ahlemeyer, Vannuruswamy Garikapati, Mohammad Rashedul Alam, Omelyan Trompak, Gani Oruqaj, Natalia El-Merhie, Michael Seimetz, Clemens Ruppert, Andreas Günther, Bernhard Spengler, Srikanth Karnati, Eveline Baumgart-Vogt

**Affiliations:** 1 Institute for Anatomy and Cell Biology, Division of Medical Cell Biology, Justus Liebig University, Aulweg 123, 35392 Giessen, Germany; 2 Department of Internal Medicine VIII, Eberhard Karls University, 72076 Tübingen, Germany; 3 Excellence Cluster Cardio-Pulmonary System, German Center for Lung Research (DZL), Universities of Giessen and Marburg Lung Center, 35392 Giessen, Germany; 4 UGMLC Giessen Biobank, Universities of Giessen and Marburg Lung Center, 35392 Giessen, Germany; 5 Center for Interstitial and Rare Lung Diseases, Department of Internal Medicine, German Center for Lung Research, Universities of Giessen and Marburg Lung Center, 35392 Giessen, Germany; 6 Institute of Inorganic and Analytical Chemistry, Justus Liebig University, 35392 Giessen, Germany; 7 Present Address: Institute for Anatomy and Cell Biology, Julius Maximilians University, 97070 Würzburg, Germany; 8 Present Address: Department of Medical Education, College of Medicine and Life Sciences, University of Toledo, Toledo, OH 43614 USA; 9 Present Address: Max Planck Institute of Molecular Cell Biology and Genetics, 01307 Dresden, Germany; 10 Present Address: Department of Internal Medicine II, Member of the German Center for Lung Research (DZL), Universities of Giessen and Marburg Lung Center (UGMLC), Justus Liebig University, 35392 Giessen, Germany; 11 Present Address: Institute for Lung Health (ILH), Member of the German Center for Lung Research (DZL), Universities of Giessen and Marburg Lung Center (UGMLC), Justus Liebig University, 35392 Giessen, Germany

**Keywords:** Catalase, Collagen, Human lung fibroblasts, Idiopathic pulmonary fibrosis, Matrix metalloproteinases, Peroxisome, PEX13, PPAR, TGF-β1

## Abstract

**Background:**

Idiopathic pulmonary fibrosis (IPF) is characterized by aberrant lung epithelial phenotypes, fibroblast activation, and increased extracellular matrix deposition. Transforming growth factor-beta (TGF-β)1-induced Smad signaling and downregulation of peroxisomal genes are involved in the pathogenesis and can be inhibited by peroxisome proliferator-activated receptor (PPAR)-α activation. However, the three PPARs, that is PPAR-α, PPAR-β/δ, and PPAR-γ, are known to interact in a complex crosstalk.

**Methods:**

To mimic the pathogenesis of lung fibrosis, primary lung fibroblasts from control and IPF patients with comparable levels of all three PPARs were treated with TGF-β1 for 24 h, followed by the addition of PPAR ligands either alone or in combination for another 24 h. Fibrosis markers (intra- and extracellular collagen levels, expression and activity of matrix metalloproteinases) and peroxisomal biogenesis and metabolism (gene expression of peroxisomal biogenesis and matrix proteins, protein levels of PEX13 and catalase, targeted and untargeted lipidomic profiles) were analyzed after TGF-β1 treatment and the effects of the PPAR ligands were investigated.

**Results:**

TGF-β1 induced the expected phenotype; e.g. it increased the intra- and extracellular collagen levels and decreased peroxisomal biogenesis and metabolism. Agonists of different PPARs reversed TGF-β1-induced fibrosis even when given 24 h *after* TGF-β1. The effects included the *reversals* of (1) the increase in collagen production by repressing *COL1A2* promoter activity (through PPAR-β/δ activation); (2) the reduced activity of matrix metalloproteinases (through PPAR-β/δ activation); (3) the decrease in peroxisomal biogenesis and lipid metabolism (through PPAR-γ activation); and (4) the decrease in catalase protein levels in control (through PPAR-γ activation) and IPF (through a combined activation of PPAR-β/δ and PPAR-γ) fibroblasts. Further experiments to explore the role of catalase showed that an overexpression of catalase protein reduced collagen production. Additionally, the beneficial effect of PPAR-γ but not of PPAR-β/δ activation on collagen synthesis depended on catalase activity and was thus redox-sensitive.

**Conclusion:**

Our data provide evidence that IPF patients may benefit from a combined activation of PPAR-β/δ and PPAR-γ.

**Supplementary Information:**

The online version contains supplementary material available at 10.1186/s12931-024-02935-7.

## Background

IPF is a severe restrictive interstitial lung disease with patient median survival of 2.5–3.5 years [1]. Concerning the pathogenesis of IPF, it is being discussed that an excessive injury response results in persistent overproduction of extracellular matrix (ECM) components by activated and proliferating fibroblasts. In addition, oxidative stress remains a major mechanism associated with the progression of this disease [2]. Today, only limited treatment options for IPF are available. Evidence-based recommendations for the pharmacological management of the disease are the tyrosine kinase inhibitor nintedanib [3, 4] and pirfenidone [4, 5], an inhibitor of TGF-β1-stimulated collagen synthesis. Both drugs increase quality of life, attenuate symptoms and slow down IPF progression, but only nintedanib influences mortality. Some of the novel medications targeted pentraxin (involved in endogenous tissue repair), lysophosphatidic acid, or connective tissue growth factor (mediates TGF-β1 downstream signaling), but failed the clinical endpoints [6, 7]. Other substances in the pipeline are nerandomilast (a tyrosine kinase inhibitor) which successfully completed phase II clinical trials [8] and inhaled treprostinil, a prostacyclin analogue. Treprostinil showed beneficial effects in the initial INCREASE trial [9] and ongoing TETON study [10] and has meanwhile been approved for the therapy of WHO group 1 pulmonary hypertension with an additional positive impact in IPF. Nevertheless, extensive research is still required to develop new therapeutic modalities.

To find therapeutic interventions for IPF, several studies explored the anti-fibrotic potentials of natural and synthetic PPAR ligands. For example, PPAR-α activation was demonstrated to attenuate fibrosis in the liver [11], heart [12] and lung [13, 14], while PPAR-β agonists exhibited anti-proliferative effects [15], but increased the secretion of TGF-β1 and ECM [16]. Ligands of PPAR-γ are most promising [17–20] and were thought to inhibit fibroblast trans-differentiation [21, 22] and to strengthen the anti-oxidative defense system [23]. In addition, pan-PPAR agonists, such as lanifibranor [24] and IVA337 [25] attenuated fibrosis. In all these studies, however, the anti-fibrotic mechanism of PPAR agonists remained unclear and was supposed to be mainly due to their anti-inflammatory activities [26]. Another drawback was the time schedule of the drug treatment. Typically, drugs were added before or together with TGF-β1, but these approaches do not reflect the patient situation where drugs can be given only after the diagnosis of the disease, years after its initiation. In two studies, PPAR-γ agonists were applied after bleomyin-induced lung injury in the mouse. Zeng et al. [27] added the PPARγ ligand asarinin 15–28 days after bleomycin administration, which reduced the severity of fibrosis. Speca et al. [22] applied GED-0507, a PPARγ modulator with strong anti-inflammatory effects, to mice on day 14 after bleomycin administration and reported resolution of fibrosis with 50% mortality rate. This post-treatment schedule reduced collagen deposition, *but to a lesser extent* than in the prevention approach used in the same study. Thus, we thought that a post-treatment with a combination of PPAR ligands may further increase the anti-fibrotic effect. Moreover, we aimed to use a human model and human cultured fibroblasts as the latter in vitro model better guaranties the drug availability and allows a selective (biochemical) analysis of changes in fibroblasts, the main players in fibrosis.

In this study, we investigated whether activation of each of the three PPARs alone or in various combinations influenced collagen synthesis and release of lung fibroblasts from control and IPF patients when given 24 h after TGF-β1, the endogenous stimulator of fibrosis. Moreover, we attempted to explore the mechanism of the anti-fibrotic effect of PPAR agonists by analyzing changes in members of matrix metalloproteinases (MMPs) [28], biogenesis and metabolism of peroxisomes [13, 14], and the protein level and activity of catalase, the major anti-oxidative enzyme in peroxisomes [29] with the highest turnover numbers of all enzymes [30].

## Methods

### Study approval

Biospecimen collection (i.e. lung tissues and fibroblasts from organ donors) was approved by the Ethics Committee of the Justus Liebig University Giessen (Az58/15 and Az111/08, JLU).

### Cell culture and drug treatment

Lung fibroblasts from control and IPF patients (Additional file: Table S1) and catalase-deficient fibroblast cell lines were cultured in Dulbecco´s Modified Eagle Medium (DMEM) with penicillin/streptomycin or puromycin, respectively. For the experiments, cells were serum-starved for 3 h, stimulated with vehicle or rhTGF-β1 for 24 h (except for Figs. 2B, C, E, 3B), followed by the addition of vehicle or drugs either alone or in combinations for another 24 h.

### Knockdown of catalase in human lung fibroblasts

Knockdown of catalase was done with CAT siRNA using ScreenFectA transfection reagent. Stable catalase knockdown was achieved by transduction with pGIPZ-shCatalase and pGIPZ-non-silencing control lentivirus vectors as described earlier [31].

### Overexpression of catalase in human lung fibroblasts

Transfection with catalase overexpression plasmid (pGL 4.14-*Catalase*) and promoter reporter plasmids *COL1A2*-luc and PPAR response element (*PPRE)*-luc were done as described earlier [13, 32]. Data from pRL-SV40 vector served to normalize results of the luciferase reporter plasmid.

### Human TGF-β1 immunoassay and sircol collagen assay

The collected culture media of control and IPF fibroblasts were used for Sircol collagen assays and TGF-β1 ELISA assay according to the manufacturers´ instructions.

### Measurements of catalase activity, hydrogen peroxide (H_2_O_2_) production and cell proliferation

Determination of catalase activity with a redox dye assay kit based on the degradation of H_2_O_2_. H_2_O_2_ produced by cultured cells was quantified using a fluorometric detection kit. The incorporation of BrdU into proliferating cells was detected with an ELISA kit. For all the aforementioned kits, we followed the manufacturers´ instructions.

### Western blotting

Proteins of total cell lysates were separated on 10% SDS-PAGE gels and blotted on polyvinylidene difluoride membranes. Specific proteins were detected using primary and horseradish peroxidase (HRP)-labelled secondary antibodies followed by chemiluminescent detection of the HRP substrate. ImageJ was used for semi-quantitative analysis of signal intensities.

### Immunofluorescence staining

Thin sections of paraffin-embedded lung tissues were incubated with primary and secondary fluorophore-labelled antibodies. Immunofluorescence images were acquired by confocal laser scanning microscopy.

### Isolation of total RNA and RT-qPCR

Total RNA was isolated using RNAzol and mRNA levels were analyzed by RT-qPCR.

### Targeted quantification of fatty acids

Arachidonic acid (AA), docosahexaenoic acid (DHA), and eicosapentaenoic acid (EPA) were analyzed in the culture medium by solid phase extraction and a targeted liquid chromatography tandem mass spectrometry (LC–MS/MS) approach as described previously [32].

### Untargeted lipidomics

Lipids were extracted from cell lysates using a biphasic methyl-*tert*-butyl ether (MTBE) extraction protocol [33] and analyzed using an untargeted LC–MS/MS method as described previously [34].

### Statistics

Analysis was done using GraphPad Prism software. Data were expressed as means ± SEM. For comparisons between two groups, the F-test was applied to compare their variances followed by Mann–Whitney U test (unequal variances) or unpaired *t*-test (equal variances). For multiple comparisons, ANOVA was used with post-hoc Tukey´s multiple comparisons test. *P* values < 0.05 were considered as statistically significant.

## Results

### Characterization of the fibrosis markers COL1 and α-SMA, as well as of PPARs in lung tissues and cultured fibroblasts from control and IPF patients

**Fig. 1:**
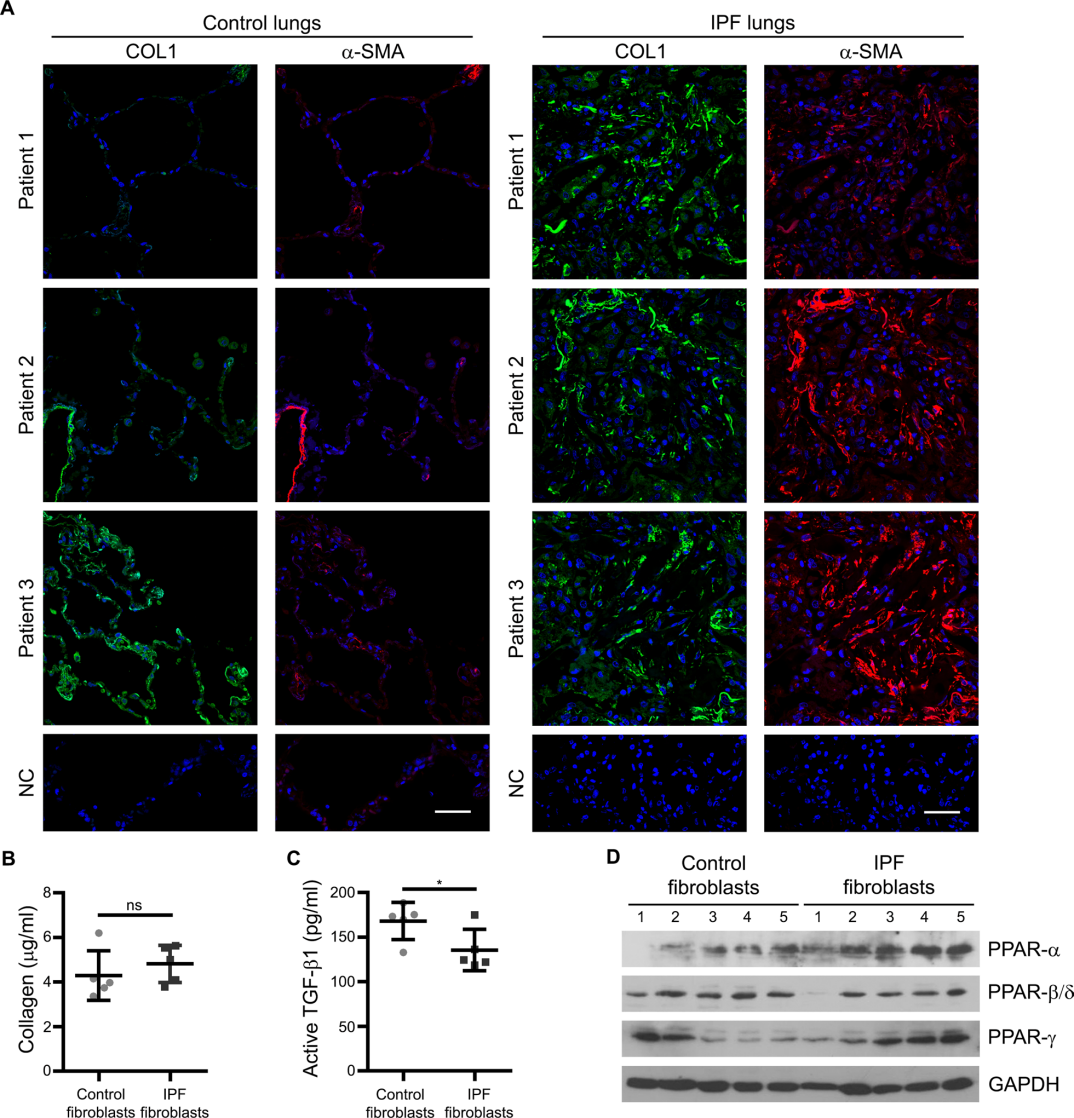
Characterization of the fibrosis markers COL1 and α-SMA, and PPARs in lung tissue and cultured fibroblasts from control and IPF patients. **A** Lung tissue sections from control (left side) and IPF (right side) patients were incubated with antibodies to detect collagen (COL1, green) and α-SMA (red), and counterstained with DAPI (blue). Negative controls (NC) were done by omitting the primary antibody*.* **B** There was no difference in the release of collagen between fibroblasts from control and IPF patients. The release of collagen into culture media was measured using Sircol assay. Data represent 5 control and 5 IPF patients across six independent fibroblast cultures. **C** The release of active TGF-β1 is higher in control than in IPF fibroblasts. The amount of active human TGF-β1 was analyzed in the culture media of fibroblasts from 5 controls and 7 IPF patients by ELISA. **D** The protein levels of PPAR-α were higher in IPF compared to control fibroblasts, whereas there was no difference with regard to PPAR-β/δ and PPAR-γ. Cultured fibroblasts from 5 control and 7 IPF patients were collected and their protein levels were analyzed by Western blot analysis with GAPDH as reference protein

The fibrosis marker collagen type I (COL1) and myofibroblast marker α-smooth muscle actin (α-SMA) were first assessed in lung biopsy samples from control and IPF patients. Lung tissues from IPF patients showed comparatively higher levels of COL1 and α-SMA than those from control subjects (Fig. 1A). Although increased mRNA levels of *COL1A1* and *ACTA2* were detected in cultured lung fibroblasts from IPF compared to control patients (Additional file: Fig. S1A, B), their protein levels and that of transforming growth factor-beta receptor 1 (TGFBR1) were higher in most cases in fibroblasts from control compared to IPF patients (Additional file: Fig. S1C, Table 1). Although unexpected at first glance, it is noteworthy that IPF lung tissue contains a much higher number of fibroblasts than controls. Probably, the higher number of fibroblasts in the lungs of IPF patients and to a minor extent their individual properties contribute to the differences in tissue pathology. Moreover, the reduced level of TGFBR1 in IPF fibroblasts suggests that they are less sensitive to TGF-β1 presumably due to their chronic exposure to the cytokine in vivo. Accordingly, analysis of extracellular collagen revealed no significant difference between control and IPF fibroblasts (Fig. 1B, Additional file: Fig. S1D). IPF is characterized by elevated levels of TGF-β1 mRNA and protein in the lung tissues of patients [35, 36]. Interestingly, the amount of released active TGF-β1 was higher in the culture media from control than IPF fibroblasts (Fig. 1C). We demonstrated an anti-fibrotic role of peroxisomes in the progression of IPF via PPAR-α signaling [13, 14]. Since all three PPARs crosstalk with each other [37], we next analyzed their protein levels in fibroblasts from control and IPF patients at basal conditions (no treatment in vitro). Collectively, IPF fibroblasts showed increased mRNA and protein levels of PPAR-α, but not of the ones of PPAR-β/δ and PPAR-γ compared to control fibroblasts (Fig. 1D, Table 1, Additional file: Fig. S1E).

**Table 1:** Densiometric analysis of the protein bands shown in Figs. 1D, 2C, D, 6C and Additional file: Fig. S1A

	Control fibroblasts		IPF fibroblasts	
		*n*		*n*
Figure 1D				
PPARα/GAPDH	1 ± 0.38	4	1.5 ± 0.10	5
PPARβ/GAPDH	1 ± 0.26	5	0.8 ± 0.05	4
PPARγ/GAPDH	1 ± 0.75	5	1.4 ± 0.60	5
Figure 2C, D				
COL1/GAPDH				
TGFβ −	1 ± 0.44	6	0.7 ± 0.52	6
TGFβ +	2.4 ± 0.74	6	1.6 ± 0.76	6
α-SMA/GAPDH				
TGFβ −	1 ± 0.36	6	0.9 ± 0.31	6
TGFβ +	1.5 ± 0.16	6	1.3 ± 0.44	6
Figure 6C				
CAT/β-ACTIN	1 ± 0.63	5	0.3 ± 0.29	5
Figure S1C				
α-SMA/β-ACTIN	1 ± 0.13	5	0.7 ± 0.46	7
COL1/β-ACTIN	1 ± 0.54	5	0.5 ± 0.62	7
TGFBR1/β-ACTIN	1 ± 0.47	5	0.3 ± 0.42	7
MMP-1/β-ACTIN	1 ± 0.72	5	1.4 ± 1.12	7

Protein band intensities of the indicated proteins normalized to the respective reference proteins were analyzed from fibroblasts of n control and IPF patients

Data from control fibroblasts were set to 1

**Fig. 2:**
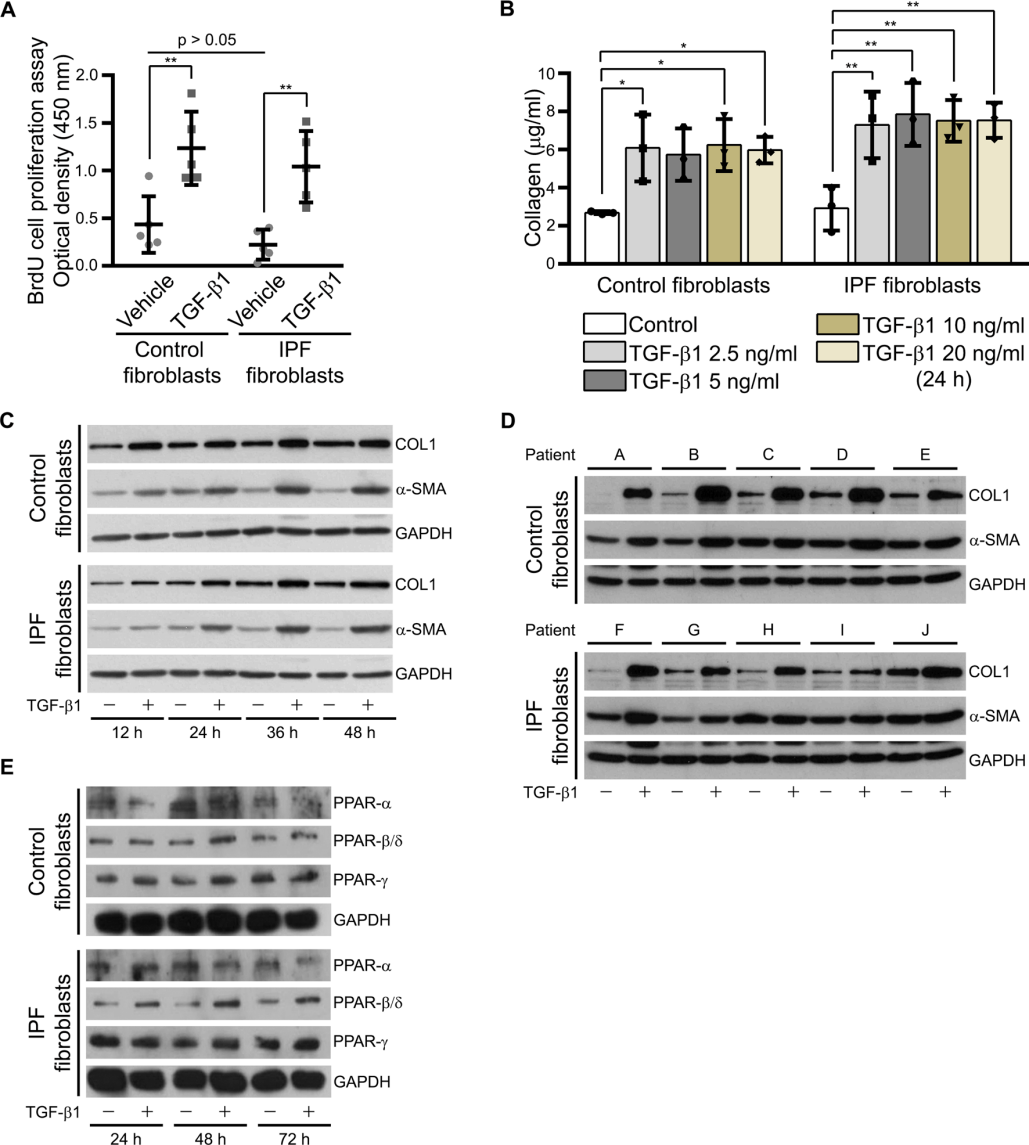
TGF-β1 induced a fibrotic response in fibroblasts from control and IPF patients. **A** TGF-β1 induced proliferation in control and IPF fibroblasts. Fibroblasts were serum-starved for 3 h and then incubated for 24 h with vehicle or TGF-β1. Thereafter, proliferation was analyzed using BrdU cell proliferation assay. **B** Treatment with different concentrations of TGF-β1 showed no difference between control and IPF fibroblasts with regard to the release of collagen into culture media. Control and IPF fibroblasts were serum-starved for 3 h and then treated with vehicle (Control) or 2.5, 5, 10 and 20 ng/ml TGF-β1 for 24 h. Cell culture media were collected and extracellular collagen was analyzed using Sircol assay. **C**, **D** TGF-β1 increased the level of intracellular COL1 in control and IPF fibroblasts in a time-dependent manner. Control and IPF fibroblasts were serum-starved for 3 h and then treated with vehicle or 5 ng/ml TGF-β1 for 12, 24, 36 and 48 h. Cell lysates were used to detect COL1 and α-SMA by Western blot analysis using GAPDH as reference protein (**C**). Data for a time period of 24 h from 5 control (patients A–E) and 5 IPF (patients F–J) patients is shown in (**D**). **E** TGF-β1 increased the protein level of PPAR-β/δ, whereas the ones of the other PPARs remained unchanged. Control and IPF fibroblasts were treated for 24, 48 and 72 h with TGF-β1 (5 ng/ml) or vehicle. Cell lysates were used for Western blot analysis of the PPARs using GAPDH as reference protein

### Activation of PPAR-β/δ induced anti-fibrotic responses in TGF-β1-stimulated fibroblasts from control and IPF patients

**Fig. 3:**
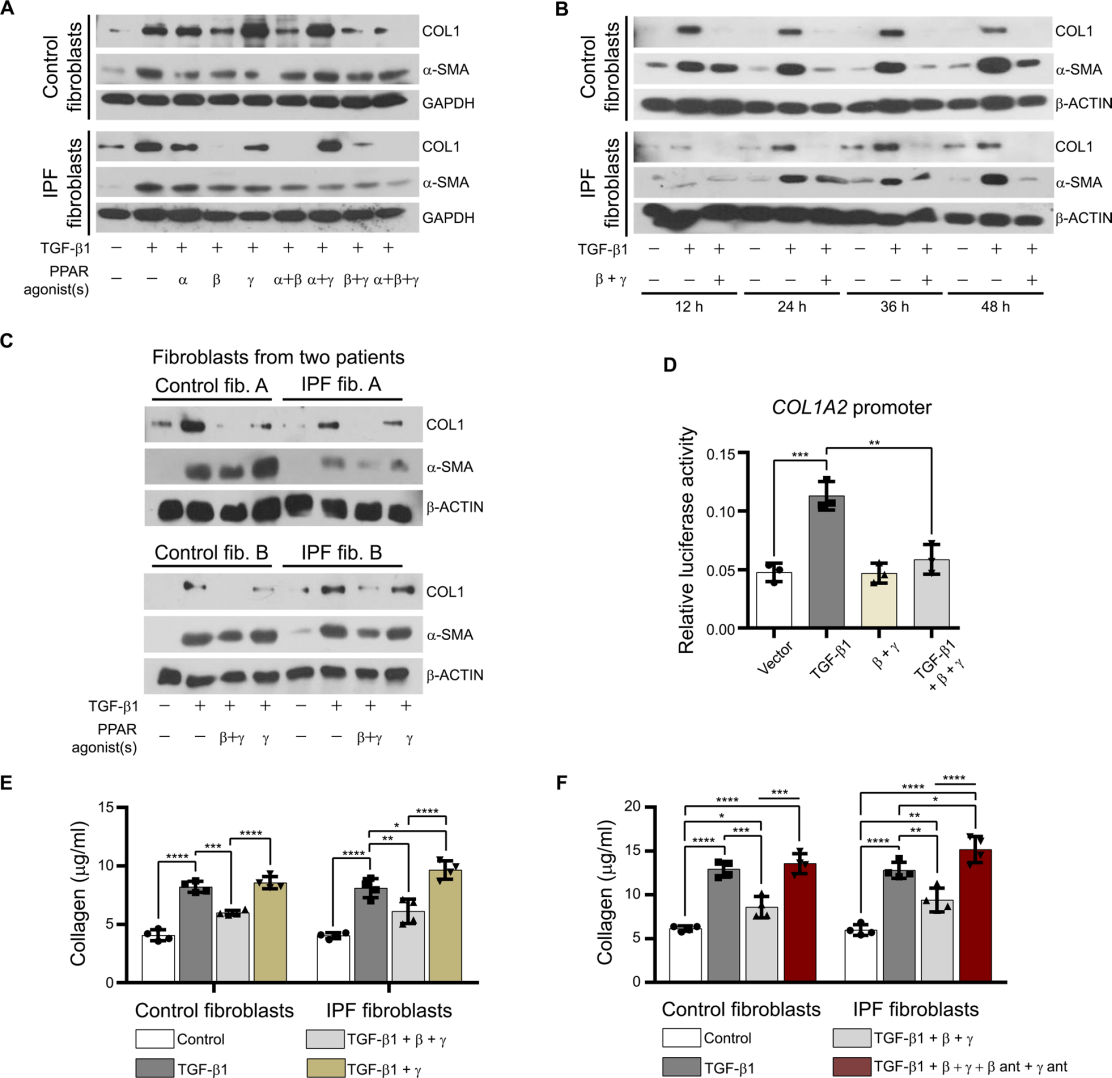
Activation of PPAR-β/δ induced anti-fibrotic responses in TGF-β1-stimulated fibroblasts. **A**–**C**,** E** Control and IPF fibroblasts were serum-starved for 3 h, treated with TGF-β1 (5 ng/ml) for 24 h, followed by the addition of the PPAR-α agonist WY14643 (100 μM, α; **A**), PPAR-β/δ agonist GW0742 (10 μM, β; **A–C**, **E**), and PPAR-γ agonist rosiglitazone (10 μM, γ; **A**–**C**,** E**) either for 24 h (**A**, **C**,** E)** or different time periods (12, 24, 36 and 48 h; **B**). **A** PPAR-β/δ activation reversed TGF-β1-induced increase in COL1. Cell lysates were used to detect COL1 and α-SMA by Western blot analysis using GAPDH as reference protein. **B**, **C** Reverse of fibrosis phenotype by PPAR-β/δ and PPAR-γ activation was stable for up to 48 h. Cell lysates at 12 to 24 h (**B**) and 48 h from two other control and IPF patients (**C**) were used for Western blot analysis using β-actin (β-ACTIN) as reference protein. **D** Combined activation of PPAR-β/δ and PPAR-γ abolished TGF-β1-induced increase in *COL1A2* promoter activity. IPF fibroblasts were transfected with a plasmid containing the luciferase firefly reporter gene adjacent to *COL1A2* promoter and Renilla luciferase as second reporter for normalization. At 72 h after transfection, cells were treated with vehicle (Vector) or TGF-β1 (5 ng/ml) for 24 h followed by the addition of the PPAR-β/δ agonist GW0742 (10 μM, β) combined with the PPAR-γ agonist rosiglitazone (10 μM, γ) or vehicle for another 24 h. Cells were lysed and collected for dual luciferase activity measurements. **E** Ligand activation of PPAR-β/δ together with PPAR-γ strongly decreased the release of collagen produced by TGF-β1-stimulation in control and IPF fibroblasts. Culture media were collected and extracellular collagen was analyzed using Sircol assay. **F** Combined activation of PPAR-β/δ and PPAR-γ decreased TGF-β1-stimulated release of collagen by control and IPF fibroblasts—this effect was blocked using the respective antagonists. Cells were serum-starved for 3 h, stimulated with vehicle (Control) or TGF-β1 (5 ng/ml) for 24 h, followed by the addition of the PPAR-β/δ agonist GW0742 (10 μM, β) and PPAR-γ agonist rosiglitazone (10 μM, γ) either combined with vehicle or the PPAR-β/δ antagonist GSK0660 (10 nM, β ant) and PPAR-γ antagonist GW9662 (10 μM, γ ant) for another 24 h. Culture media were collected and extracellular collagen was analyzed by Sircol assay

As already noted, the number of fibroblasts in the lungs of IPF patients might be crucial for the disease progression. To confirm this, we analyzed the proliferation of vehicle- and TGF-β1-treated control and IPF fibroblasts since the cytokine was used to mimic part of the disease condition in vitro. As expected, TGF-β1 stimulated cell proliferation in control and IPF fibroblasts (Fig. 2A). Next, we analyzed time-dependent changes in α-SMA and COL1 protein levels of control and IPF fibroblasts treated with TGF-β1. Control and IPF fibroblasts did not show differences after stimulation with different concentrations of TGF-β1 (2.5–20 ng/ml; Fig. 2B) in the extracellular collagen released into the culture media. Though 2.5 ng/ml of TGF-β1 was already sufficient to reach the maximal effect for collagen values 24 h after treatment (Fig. 2B), 5 ng/ml TGF-β1 was used to obtain maximal effects in all following experiments with distinct parameters. TGF-β1 increased intracellular COL1 and α-SMA protein levels from 12 to 48 h in control fibroblasts and from 24 h up to 48 h in IPF fibroblasts (Fig. 2C). Moreover, the treatment with TGFβ-1 for 24 h in control and IPF fibroblasts from 10 different patients showed a homogenous and stable increase in the protein levels of COL1, but an inconsistent reaction in the case of α-SMA (Fig. 2D, Table 1). To investigate the role of peroxisomes in IPF, their proliferation was induced using different PPAR ligands. Interestingly, TGF-β1 upregulated the protein level of PPAR-β/δ especially after 48 h of treatment (Fig. 2E). Following 24 h TGF-β1 stimulation, treatment with PPAR-β/δ agonist alone or in combination with the two other members of the PPAR protein family inhibited the TGF-β1-mediated increase in COL1 and—to a lesser extent—α-SMA protein levels in control and IPF fibroblasts (Fig. 3A). As already noted, anti-fibrotic properties of PPAR-γ have been reported in the past. In our study, the post-treatment with a PPAR-β/δ agonist (GW0742) alone or combined with a PPAR-γ agonist (rosiglitazone) strongly decreased the amount of TGF-β1-mediated increase in intracellular COL1 (Fig. 3A–C) by affecting *COL1A2* promotor activity (Fig. 3D) as well as extracellular collagen (Fig. 3E) in both, fibroblasts from control and IPF patients. Lesser effects were observed in the case of activation of PPAR-γ alone (Fig. 3A, C, E). The decrease in the amount of COL1 as a result of the dual treatment of PPAR-β/δ and PPAR-γ agonists was stable over time (Fig. 3B) and between patients (Fig. 3C). Furthermore, the anti-fibrotic effects of a combined activation of PPAR-β/δ and PPAR-γ were blocked in the presence of PPAR-β/δ (GSK0660) and PPAR-γ (GW9662) antagonists (Fig. 3F). In addition, we thought to use the compound STK 648389 (ZINC ID: 31,775,965), a putative dual agonist for PPAR-β/δ and PPAR-γ. However, analysis of the STK 648389 for its effect on collagen showed adverse effects and even increased extracellular collagen levels released by control and IPF fibroblasts after TGF-β1 exposure (Additional file: Fig. S2). Altogether, these findings suggest that although TGF-β1 increases the PPAR-β/δ protein as a protective adaptive mechanism, endogenous PPAR-β/δ activating ligands are probably diminished to prevent fibrosis in patients.

### PPAR-β/δ triggers anti-fibrotic responses by activating MMP-1 in control and IPF fibroblasts

**Fig. 4:**
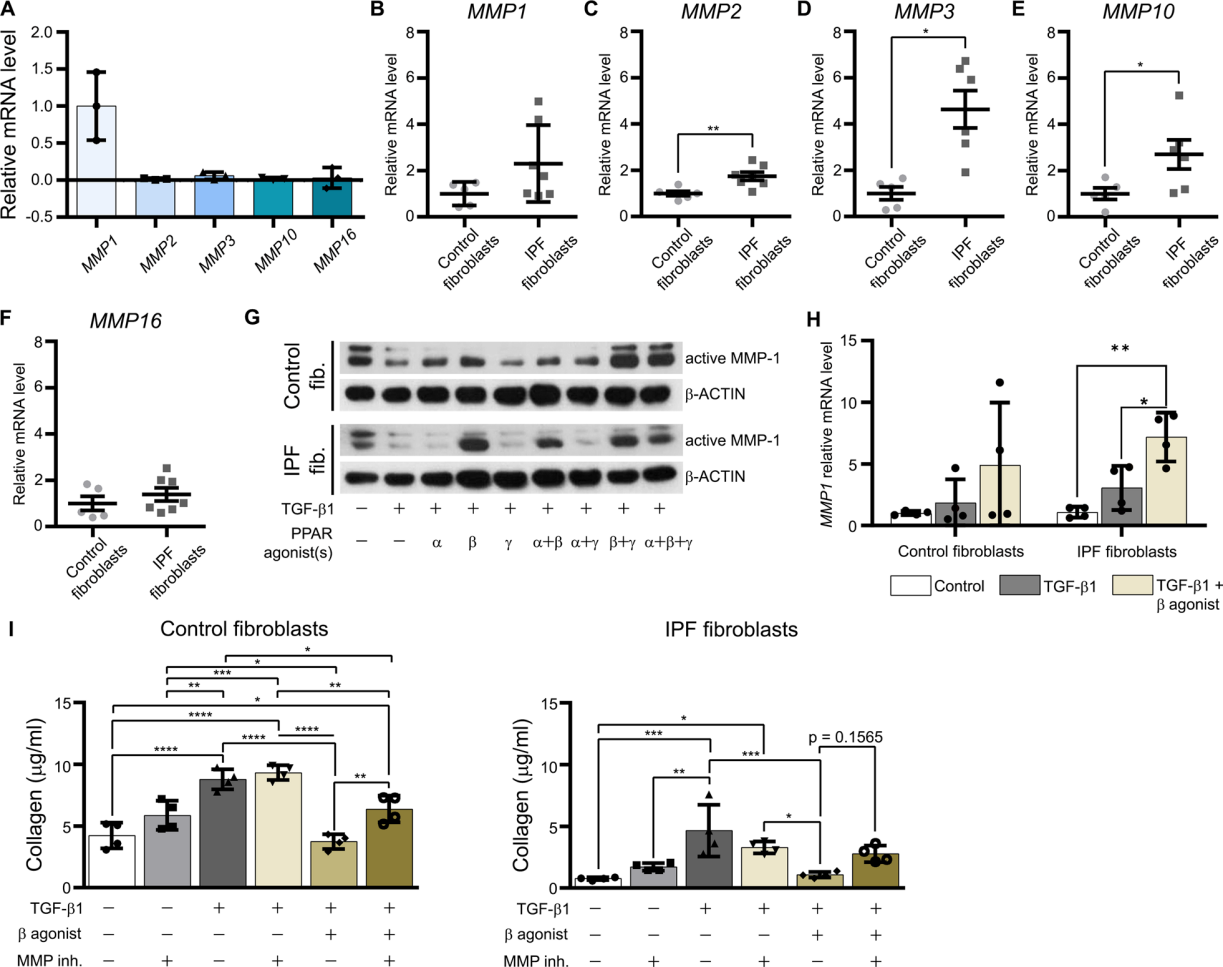
PPAR-β/δ triggers anti-fibrotic responses by activating MMP-1 in control and IPF fibroblasts. **A** The transcript of *MMP1* is the highest among the different *MMPs* in control fibroblasts. Analysis of *MMP1, MMP2, MMP3, MMP10* and *MMP16* of control fibroblasts was done using isolated total RNA and RT-qPCR with *HPRT1* as reference gene. **B**–**F** Comparative gene expression profile of *MMPs* was done by RT-qPCR with *HPRT1* as reference gene. **G** PPAR-β/δ attenuated TGF-β1-induced decrease in the amount of active MMP-1. Control and IPF fibroblasts were serum-starved for 3 h, treated with vehicle or TGF-β1 (5 ng/ml) for 24 h, followed by the addition of the PPAR-α agonist WY14643 (100 μM, α), PPAR-β/δ agonist GW0742 (10 μM, β), and PPAR-γ agonist rosiglitazone (10 μM, γ) as well as various combinations thereof for another 24 h. Cell lysates were used to detect active MMP-1 by Western blot analysis using β-actin (β-ACTIN) as reference protein. **H** Ligand activation of PPAR-β/δ strongly increased the mRNA level of *MMP1* in TGF-β1-treated control and IPF fibroblasts. Cells were serum-starved, treated with vehicle (Control) or TGF-β1 (5 ng/ml) for 24 h followed by the addition of the PPAR-β/δ agonist GW0742 (10 μM, β) or vehicle for another 24 h. The mRNA levels were measured by RT-qPCR with *HPRT1* as reference gene. **I** Inhibition of MMPs increased TGF-β1-induced release of collagen. Control and IPF fibroblasts were serum-starved for 3 h, treated with vehicle or TGF-β1 (5 ng/ml) for 24 h, followed by the addition of the PPAR-β/δ agonist GW0742 (10 μM, β) and MMP inhibitor (MMP inh., 4-aminobenzoyl-Gly-Pro-D-Leu-D-Ala hydroxamic acid, 20 μM) for another 24 h. The release of collagen into the culture media was measured by Sircol assay

Extracellular collagen is degraded by proteinases, e.g. MMPs. The mRNA levels of selected MMPs in control fibroblasts at basal condition (without treatment) were measured, showing the highest value for *MMP1* in comparison to the lower mRNA values for *MMP2*, *MMP3*, *MMP10*, and *MMP16* (Fig. 4A). Interestingly, the mRNA level of *MMP7* which is associated with disease severity [28] was below detectable levels in our samples of control and IPF fibroblasts (ct values > 35). Comparing the mRNA levels between control and IPF fibroblasts, no differences were observed in the case of *MMP1* and *MMP16* (Fig. 4B, F), but higher levels were found for *MMP2*, *MMP3* and *MMP10* (Fig. 4C–E). Individual mRNA values for *MMP1*, but also for *MMP3* and *MMP10*, varied strongly within the IPF sample group (Fig. 4B, D, E). Due to the much higher mRNA levels for *MMP1* compared to the other *MMPs* (Fig. 4A), we analyzed MMP-1 protein as the dominant enzyme for collagen degradation in subsequent experiments. As expected, the protein level of active MMP-1 was reduced by TGF-β1 and restored in the presence of PPAR-β/δ agonist alone or in combination with PPAR-α or PPAR-γ agonists (Fig. 4G). This suggests that PPAR-β/δ might be a key regulator of the protein level of active MMP-1. Therefore, we analyzed the effect of the PPAR-β/δ agonist in TGF-β1-stimulated fibroblasts at the mRNA levels of all detectable *MMPs*. The mRNA levels of *MMP1* in IPF fibroblast were increased (> fivefold) by the PPAR-β/δ agonist in comparison to TGF-β1 stimulation alone (Fig. 4H). The *MMP16* mRNA levels were elevated > fivefold in both types of fibroblasts and that of *MMP10* about threefold in control fibroblasts only (Additional file: Fig. S3). To explore the anti-fibrotic potential of increased levels of MMP*s*, we used a broad-spectrum inhibitor for MMPs, primarily influencing the amount of extracellular collagen. Simultaneous treatment with the MMP inhibitor and PPAR-β/δ agonist after TGF-β1 stimulation increased extracellular collagen in the culture media released by control fibroblasts, but not in the case of IPF fibroblasts (Fig. 4I). Since the MMP inhibitor only partly blocked the effect of the PPAR-β/δ agonist, we speculate that activated PPAR-β/δ also regulates other proteins involved in fibrosis attenuation.

### Activation of PPAR-β/δ and PPAR-γ in TGF-β1-treated fibroblasts increased peroxisomal biogenesis and lipid metabolism, and the inhibited fibrotic response

**Fig. 5:**
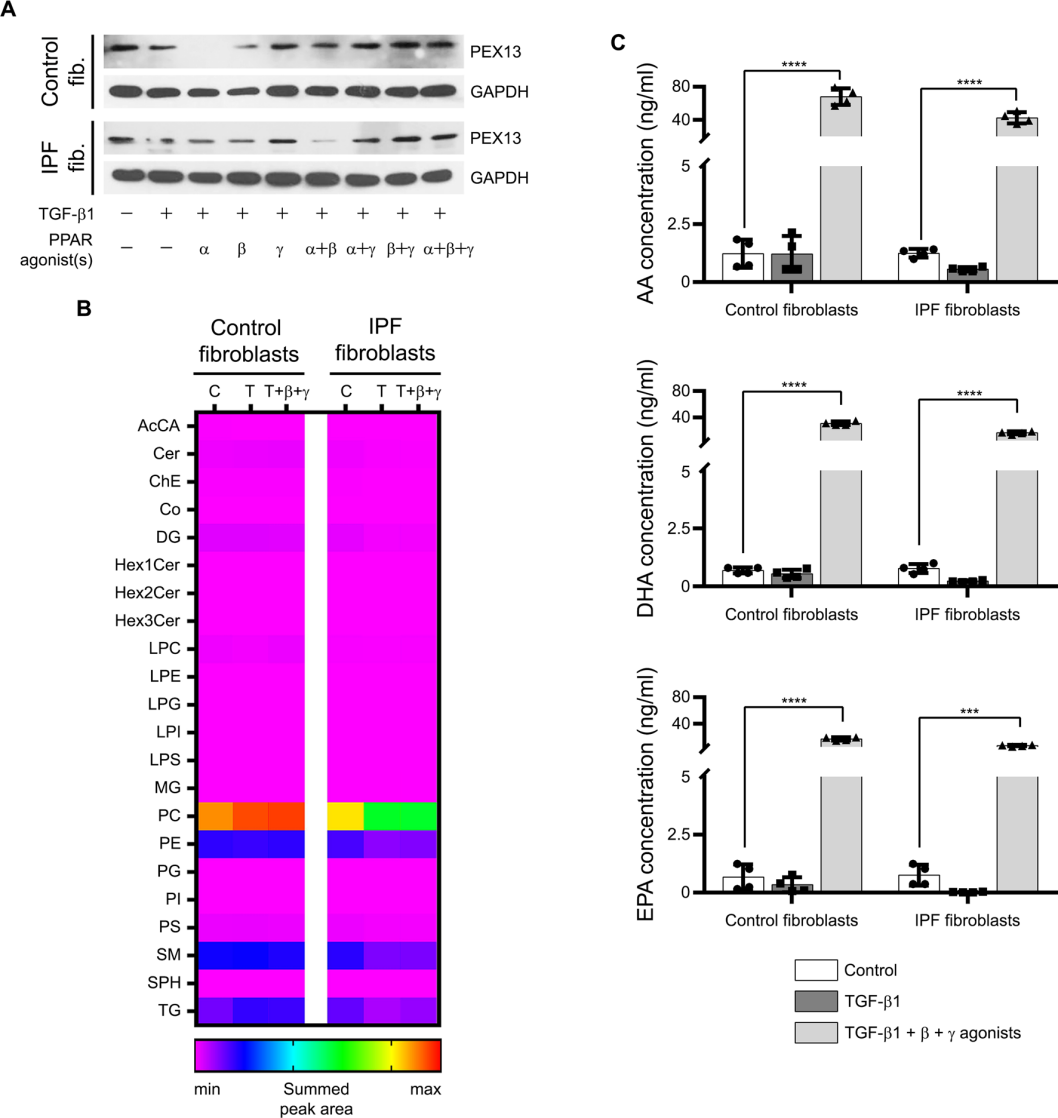
Activation of PPAR-β/δ and PPAR-γ in TGF-β1-treated fibroblasts increased peroxisomal biogenesis and lipid metabolism. **A**-**C** Control and IPF fibroblasts were serum-starved for 3 h, treated with vehicle or TGF-β1 (5 ng/ml) for 24 h, followed by the addition of the PPAR-α agonist WY14643 (100 μM, α; **A**), PPAR-β/δ agonist GW0742 (10 μM, β; **A**–**C**), and PPAR-γ agonist rosiglitazone (10 μM, γ; **A-C**) as well as various combinations thereof for another 24 h. **A** Activation of PPAR-β/δ and PPAR-γ reversed TGF-β1-induced decrease in the protein levels of the peroxisomal biogenesis protein PEX13. Cell lysates were used for Western blot analysis of PEX13 using GAPDH as reference protein. **B** Heatmap of the lipidomic profile of control and IPF fibroblasts. Cells were collected in PBS for lipid analysis using LC–MS/MS. **C** Activation of PPAR-β/δ and PPAR-γ increased the synthesis of endogenous activators of these receptors in line with a positive feedback loop. Fibroblasts from control and IPF patients were serum-starved for 3 h, treated with vehicle (Control) or TGF-β1 (5 ng/ml) for 24 h, followed by the addition of vehicle or the PPAR-β/δ agonist GW0742 (10 μM, β) combined with the PPAR-γ agonist rosiglitazone (10 μM, γ) for another 24 h. The releases of AA, DHA, and EPA were analyzed in the culture media by LC–MS/MS

Previously, we showed that pretreatment with PPAR-α agonists could inhibit fibrosis phenotypes [13, 14]. In the present study, we treated control and IPF fibroblasts with TGF-β1 before the addition of agonists of all three PPARs, an experimental setup that more accurately recapitulates the clinical setting. We first investigated the mRNA levels of several peroxisomal genes involved in the organelle biogenesis (*PEX13, PEX14*), plasmalogen synthesis (*AGPS, GNPAT*), and fatty acid β-oxidation (*ACOX1, ACAA1*) in control and IPF fibroblasts. The mRNA levels of *PEX13*, *ACOX1* and *AGPS* were higher in IPF compared to control fibroblasts, whereas those of *PEX14, ACAA1* and *GNPAT* were not significantly different (Additional file: Fig. S4A–F). Next, we stimulated peroxisomal proliferation with different PPAR agonists (alone or in combination) in TGF-β1-treated control and IPF fibroblasts. Combined activation of PPAR-β/δ and PPAR-γ increased mRNA (Additional file: Fig. S4G) and protein levels (Fig. 5A) of PEX13 in TGF-β1-stimulated control and IPF fibroblasts compared to TGF-β1 treatment only. Since the combined activation of PPAR-β/δ and PPAR-γ reversed the TGF-β1-induced trans-differentiation of fibroblasts into myofibroblasts (as indicated by changes in the level of α-SMA, Fig. 3A–C), decreased the protein level of COL1 (Fig. 3A–C) and increased PEX13 (Fig. 5A), we focused on these two PPAR agonists in the following experiments. First, the intracellular lipidomic profile was assessed in control and IPF fibroblasts to ascertain possible differences in the lipid metabolism under basal conditions and after treatments with TGF-β1 alone and PPAR-β/δ and PPAR-γ agonists. In total, 1003 lipid ion species covering 5 major lipid categories (glycerophospholipids, sphingolipids, glycerolipids, fatty acyls, and sterols) belonging to 22 lipid classes were identified based on high mass accuracy (5 ppm) and their fragmentation patterns (Additional file: Fig. S5A). Basal levels of all classes of lipids analyzed were lower in IPF fibroblasts except for the triglycerides (TG; Fig. 5B). TGF-β1 decreased the levels of phosphatidylcholine (PC), phosphatidylethanolamine (PE), sphingomyelin (SM) and TG in IPF fibroblasts. The levels of PC, SM and TG were partially restored by a post-treatment with PPAR-β/δ and PPAR-γ agonists (Fig. 5B). Furthermore, activation of PPAR-β/δ and PPAR-γ strongly increased the synthesis of peroxisome-derived AA, DHA, and EPA (Fig. 5C), which are all endogenous activators of PPARs. In the absence of TGF-β1, PPAR agonists either increased or decreased the levels of PC in control and IPF fibroblasts (Additional file: Fig. S5B) and increased the levels of AA, DHA and EPA with PPAR-γ exhibiting the strongest effect on DHA (Additional file: Fig. S5C). This suggests that, the PPAR-γ agonist was the driving factor for the increase and release of AA, DHA and EPA in fibroblasts treated with TGF-β1 followed by combined PPAR-β/δ and PPAR-γ agonists treatment (Fig. 5C). Collectively, activation of PPAR-β/δ and PPAR-γ potentially regulates the fibrosis phenotype by modulating peroxisomal lipid metabolism, but differently in control and IPF fibroblasts.

### Activation of PPAR-β/δ in combination with PPAR-γ restored TGF-β1-induced decrease in catalase mRNA and protein levels

**Fig. 6:**
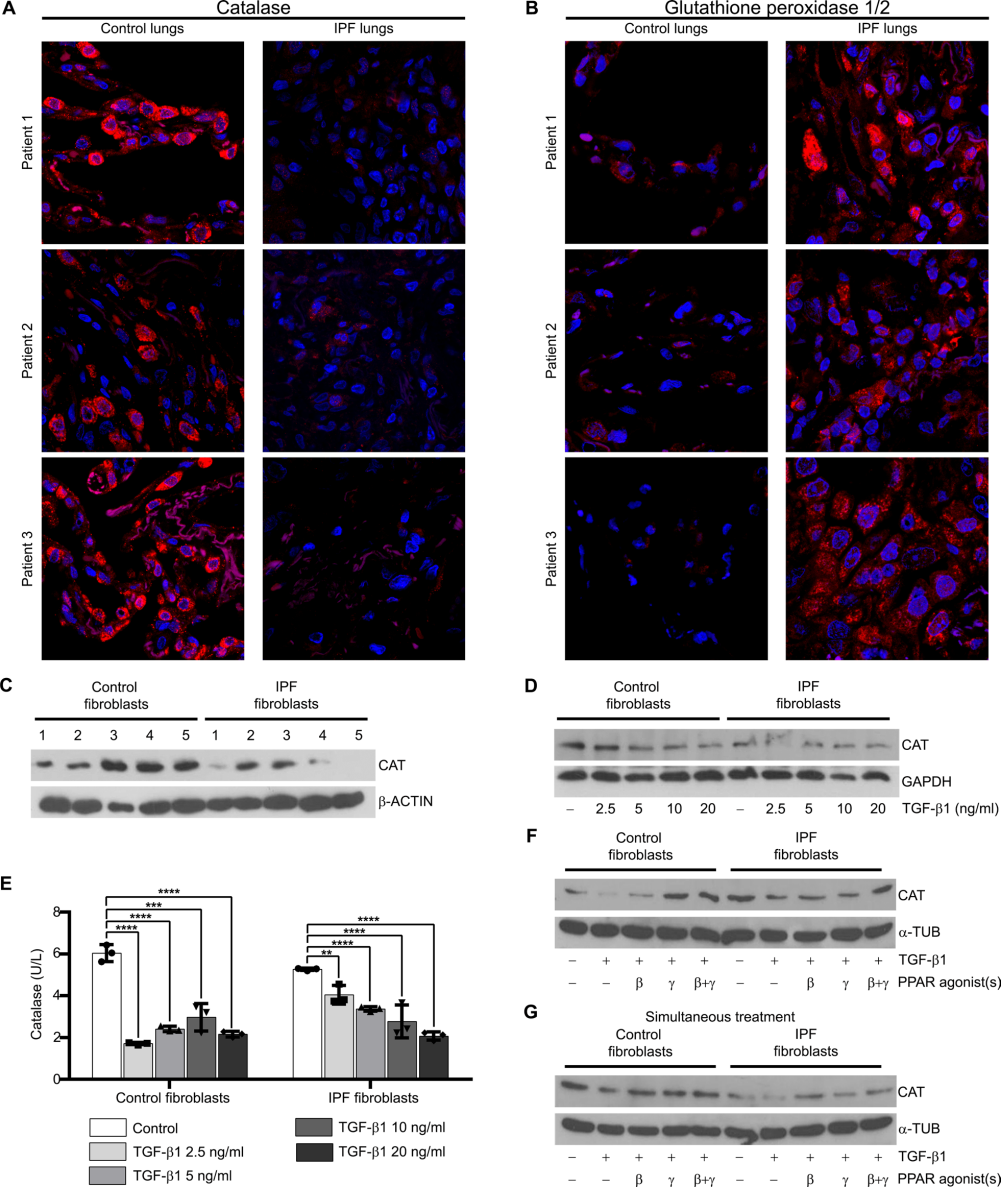
TGF-β1 caused a decrease in catalase mRNA and protein levels. **A**, **B** The immunoreactivity of catalase was lower, and that of GPX1/2 higher in IPF (right) compared to control (left) lung tissues. Immunofluorescence staining was performed using antibodies to detect catalase (**A**, red) and GPX1/2 (**B**, red) and DAPI to counterstain nuclei. **C** The protein level of catalase is lower in IPF than in control fibroblasts. Cell lysates of fibroblasts from 5 control and 5 IPF patients were used for Western blot analysis of catalase (CAT) with β-actin (β-ACTIN) as reference protein. **D** TGF-β1 decreased catalase protein levels in control and IPF fibroblasts. Cells were serum-starved for 3 h, and treated with various concentrations of TGF-β1 or vehicle for 48 h. Cell lysates were used for Western blot analysis of catalase with GAPDH as reference protein. **E**–**G** Activation of PPAR-β/δ in combination with PPAR-γ restored TGF-β1-induced decrease in catalase protein levels and activity. **E** TGF-β1 decreased catalase activity in control and IPF fibroblasts. Cells were serum-starved for 3 h, and treated with vehicle (Control) or various concentrations of TGF-β1 for 12 h. Cell lysates were used for measuring catalase activity. **F**, **G** Activation of PPAR-β/δ in combination with PPAR-γ inhibited TGF-β1-induced decrease in catalase protein levels in control and IPF fibroblasts. Cells were serum-starved for 3 h, stimulated with vehicle (**F**, **G**) or TGF-β1 (5 ng/ml, **F**, **G**) or for 24 h, followed by the addition of the PPAR-β/δ agonist GW0742 (10 μM, β) and the PPAR-γ agonist rosiglitazone (10 μM, γ) for another 24 h (**F**). In (**G**), the PPAR agonists were added together with TGF-β1 for 48 h. Cell lysates were used to detect catalase (CAT) by Western blot analysis using α-tubulin (α-TUB) as reference protein

Though not significant, TGF-β1 decreased *CAT* mRNA level in control and IPF fibroblasts, which was restored by the combined activation of PPAR-β/δ and PPAR-γ (Additional file: Fig. S4G). We therefore speculated that this anti-oxidative enzyme might be involved in regulation of fibrogenesis. We first analyzed catalase and glutathione peroxidase (GPX)1/2 in human lung tissue samples. The protein level of catalase was markedly decreased in alveolar epithelial type II cells in the lungs of IPF compared to control patients (Fig. 6A), whereas that of GPX1/2 was increased (Fig. 6B), probably to compensate catalase deficiency. Moreover, we detected a gradual decrease in catalase protein level in mouse lungs after bleomycin-induced fibrosis, remarkably from day 14 after treatment (Additional file: Fig. S6A). When we analyzed the fibroblasts from control and IPF patients, we found no differences in the mRNA levels of *CAT* and *GPX1/2* (Additional file: Fig. S6B, C). Protein level of catalase was lower in IPF compared to control fibroblasts (isolated from 5 patients each, Fig. 6C, Table 1). Apart from catalase and GPX1/2, peroxiredoxins (PRDXs) were measured as they also support the anti-oxidant defense system. The mRNA levels of different peroxiredoxin family members varied strongly (Additional file: Fig. S6D) with *PRDX6* showing the highest and PRXD2 and PRXD3 the lowest gene expression levels. Only the mRNA levels of *PRDX4* and *PRDX6* were significantly higher in IPF compared to control fibroblasts (Additional file: Fig. S6E–J). To confirm the regulatory effects of TGF-β1 on catalase, we treated control and IPF fibroblasts with TGF-β1 at various concentrations. Increasing concentrations of TGF-β1 gradually decreased the protein level of catalase in both fibroblast groups (Fig. 6D). Catalase activity was reduced by TGF-β1 in control and IPF fibroblasts, but not in the same manner since IPF fibroblasts were less sensitive towards lower concentrations of TGF-β1 (2.5 and 5 ng/ml; Fig. 6E). Activation of PPAR-γ increased the protein level of catalase in the absence of TGF-β1 (Additional file: Fig. S6K) and reversed the TGF-β1-induced decrease in catalase in control fibroblasts (Fig. 6F). The level of catalase increased in *both* groups when PPAR-β/δ and PPAR-γ were activated 24 h after TGF-β1 treatment (Fig. 6F), but not when added together with TGF-β1 (Fig. 6G).

### Catalase contributes to collagen reduction in pulmonary fibrosis

**Fig. 7:**
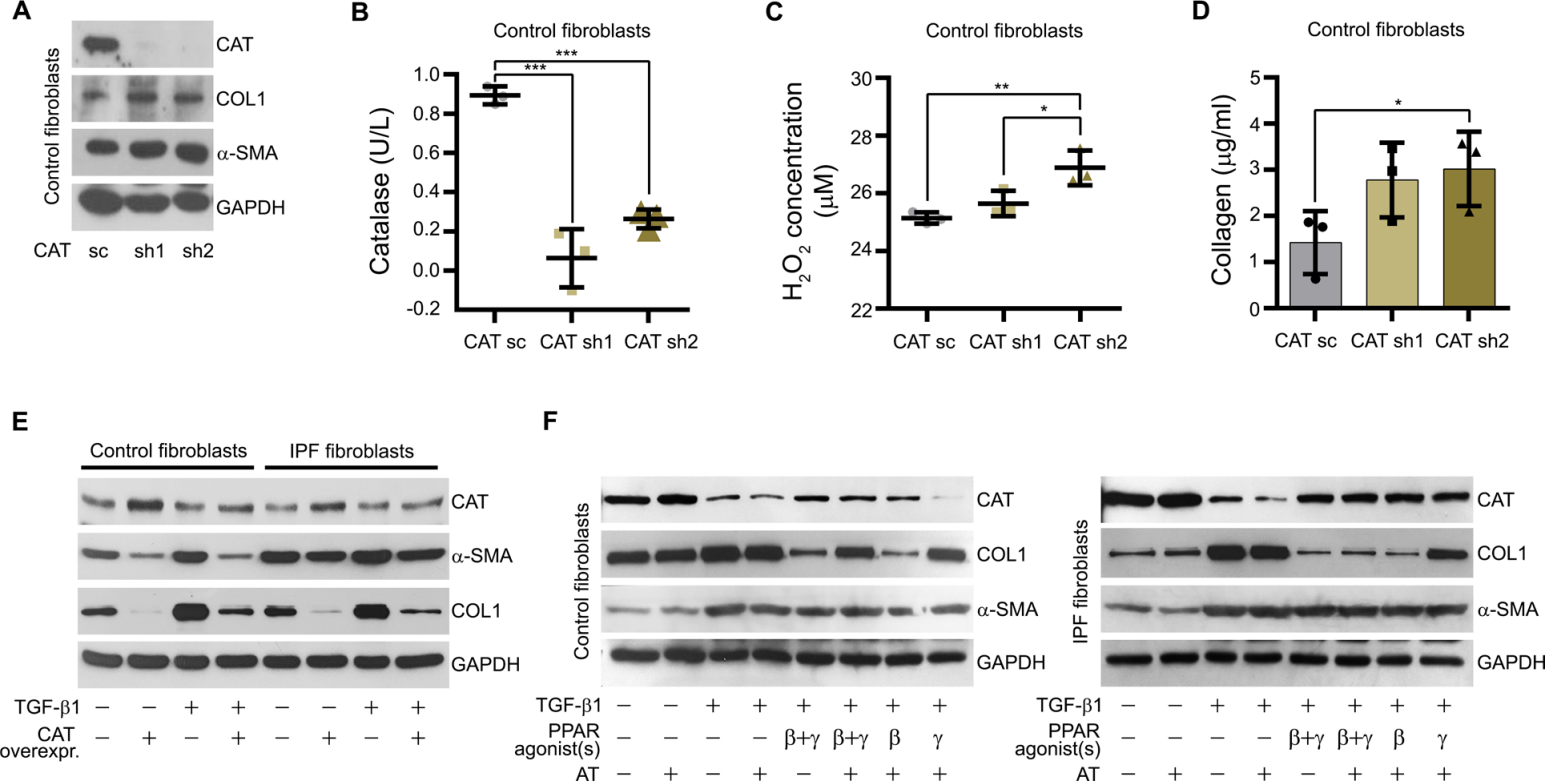
Catalase contributes to collagen reduction in pulmonary fibrosis. **A**, **B** Stable knockdown of catalase decreased catalase protein and activity. Cell lines transfected with catalase shRNA (CAT sh1, CAT sh2) were serum-starved for 3 h. Cell lysates were used for measuring catalase (CAT), COL1 and α-SMA protein levels by Western blot analysis using GAPDH as reference protein (**A**) and catalase activity by catalase activity assay kit (**B**). **C**, **D** Stable knockdown of catalase increased the cellular H_2_O_2_ production and extracellular collagen levels. Culture media from catalase-deficient (CAT sh1, CAT sh2) and mock-transfected (CAT sc) control fibroblasts were used to detect the release of H_2_O_2_ using the hydrogen peroxide assay (**C**) and of extracellular collagen by Sircol assay (**D**). **E** Overexpression of catalase decreased the protein level of COL1 in control and IPF fibroblasts under basal condition (no treatment) and after TGF-β1 treatment. Control and IPF fibroblasts were transfected with pGL 4.14-Catalase (CAT overexpr.) or a mock vector for 48 h, followed by the addition of vehicle or TGF-β1 (5 ng/ml) for another 48 h. Cell lysates were analyzed for catalase (CAT), α-SMA, and COL1 protein levels by Western blot analysis using GAPDH as reference protein. **F** The catalase activity inhibitor AT does not increase COL1 in control and IPF fibroblasts. Cells were serum-starved for 3 h, treated with vehicle or TGF-β1 (5 ng/ml) or for 24 h, followed by the addition of the PPAR-β/δ agonist GW0742 (10 μM, β), the PPAR-γ agonist rosiglitazone (10 μM, γ) and AT (25 µM) as well as various combinations thereof for another 24 h. Cell lysates were used to analyze catalase (CAT), COL1, and α-SMA protein levels by Western blot analysis using GAPDH as reference protein

To confirm the anti-fibrotic role of catalase in IPF, we intended to generate stable catalase-deficient fibroblast cell lines by RNAi using two independent shRNAs against catalase (CAT sh1 RNA and CAT sh2 RNA). Knockdown efficiency of catalase was high and stable in control fibroblasts, whereas IPF fibroblasts died after a few passages probably because the catalase protein level was already low prior to shRNA transduction (see Fig. 6C) and a further decrease in this protein was detrimental. Successful reduction of catalase is shown on the protein (Fig. 7A) and activity (Fig. 7B) levels, resulting in an increase in H_2_O_2_ concentration (Fig. 7C). The decrease in catalase protein in control fibroblasts expressing either of the two independent catalase shRNAs was accompanied with increased extracellular collagen (Fig. 7D) and intracellular COL1 (Fig. 7A) levels. Using siRNA technology, a transient catalase knockdown was achieved in control and IPF fibroblasts (Additional file: Fig. S7A). In IPF fibroblasts, we detected higher levels of collagen released into the culture medium compared to those transfected with scrambled control siRNA (Additional file: Fig. S7B). Moreover, catalase overexpression in control and IPF fibroblasts decreased COL1 and α-SMA protein levels even after TGF-β1 stimulation (Fig. 7E). Lastly, we analyzed whether the reduction in collagen synthesis by activation of PPAR-β/δ and PPAR-γ depends on catalase activity. In both fibroblast cell lines, the reduction in collagen by the PPAR-γ agonist, but not by PPAR-β/δ was reversed in the presence of 3-amino-1,2,4-triazole (AT, Fig. 7F, lane 5 versus lanes 7 and 8). Interestingly, AT inhibited the beneficial effect of a combined activation of PPAR-β/δ and PPAR-γ in control, but not in IPF fibroblasts (Fig. 7F, lane 5 versus lane 6). We suggest that during TGF-β1 treatment either the protein level, sensitivity or signaling of PPAR-β/δ dominates in IPF and that of PPAR-γ in control fibroblasts with regard to catalase protein content and its activity.

## Discussion

In the present study, cultured human lung fibroblasts were treated with TGF-β1 to mimic fibrosis and were then analyzed to evaluate the role of PPARs during disease progression. Human lung tissue samples from control and IPF patients (Figs. 1, 6) were used in parallel. Traditional animal models of experimental lung fibrosis were carried out by radiation or intratracheal administration of asbestosis fibers and silica, but the latter two induce rather asbestosis and silicosis than fibrosis [38]. Since high levels of TGF-β1 were shown to initiate and support fibrosis [35, 36], a rat model of adenoviral overexpression of TGF-β1 has been established, however, the adenovirus vector itself already induced fibrosis [38, 39]. Most commonly, mice were treated with bleomycin which induced a rapid fibrosis within 2–4 weeks via intra-tracheal instillation or 4–12 weeks by systemic administration [38]. The injury first triggers an inflammatory response which leads to wound healing. The infiltrating immune cells produce pro-fibrotic cytokines, e.g. TGF-β1, which stimulates fibroblast-to-myofibroblast transition. A dysregulated wound healing process could moreover lead to excessive deposition of ECM and finally resulting in fibrosis. However, this mouse model does not represent all aspects of the histopathological phenotype of the disease as observed in humans, for example, honeycomb pattern, thick scars at the alveolar region and fibroblastic foci [40–42], probably because these features take time to develop in humans. In addition, bleomycin-induced fibrosis is often reversible and contains a strong inflammatory component in the beginning which is not true for the disease in humans [38].

To mimic fibrosis in vitro, pro-fibrotic cytokines were added to cultured lung fibroblasts such as platelet-derived growth factor, connective tissue growth factor, interleukin-1β, tumor necrosis factor-α (TNF-α) and TGF-β1 [43]. Interleukin-1β and growth factors induced a marked inflammation and fibrosis with aberrant wound healing, TNF-α induced a strong inflammation and mild fibrosis, and TGF-β1 solely caused minor inflammation together with a marked fibrosis. Thus, TGF-β1-induced changes reflected the pathogenesis found in human IPF patients and was therefore used in our experiments. In vitro models, as an advantage, allow drug treatments to block TGF-β1-induced fibrosis signaling pathways and cell transfection to knockdown proteins of interest, which is difficult to establish in vivo. On the other hand, analysis of cultured lung fibroblasts neglects the in vivo situation where they interact with themselves and other cell types such as alveolar epithelial cells type I and type II, endothelial cells and macrophages. Interestingly, alveolar epithelial type II cells restrict the number of fibroblasts [44], and thus, control fibroblasts in vitro (and in the absence of alveolar epithelial type II cells) might re-start proliferation together with an increased collagen synthesis reaching similar levels as found in IPF fibroblasts. Moreover, TGF-β1 in IPF is mainly produced by macrophages [45]. Therefore, TGF-β1 (at least 5 ng/ml) had to be added to induce fibrosis in cultures of pure fibroblasts (which secrete 0.15 ng/ml TGF-β1, Fig. 1C). In this study, tissues and an in vitro model established with fibroblasts from control and IPF patients were used in parallel.

To study the pathophysiology of lung fibrosis, we measured the two fibrosis markers associated with IPF such as collagen [46–48], and α-SMA, although the latter has been currently debated as a sole marker for studying fibrosis [49] as its expression doesn´t mean that a cell produces high amounts of collagen [50]. Interestingly, IPF is characterized by excessive accumulation of collagen-rich ECM produced by activated fibroblasts and myofibroblasts [51, 52]; thus the degree of fibrosis is strongly dependent on their number and proliferation. Our data showed that fibroblasts from control and IPF patients were not different with regard to (1) the intracellular level of α-SMA and ω-fatty acids such as AA, DHA and EPA; (2) the release of collagen into the extracellular space; (3) the activity of collagen-degrading enzyme MMP-1; and (4) cell proliferation rate under basal conditions. Instead, fibroblasts from IPF compared to control patients showed significantly lower protein levels of PEX13, catalase, and of the TGFBR1 and are thus less sensitive towards TGF-β1. They secrete less active TGF-β1 into the culture medium. Contrarily, higher protein levels were found in IPF compared to control fibroblasts for intracellular GPX1/2 and PPAR-α. For IPF, the number and proliferation of fibroblasts/myofibroblasts are directly and the level of catalase indirectly related to the disease progression. Nonetheless, individual fibroblasts from control and IPF patients differ strongly even within the group (Figs. 1D, 6C*,* Additional file: Fig. S1A–C). This phenomenon might probably be due to the recently reported spatial heterogeneity of fibroblasts in fibrotic foci containing multiple subtypes such as lipofibroblasts, myofibroblasts, EBF1 + fibroblasts, intermediate fibroblasts, and mesothelial cells, all expressing different amounts of collagen under healthy conditions and during IPF progression [50]. In addition, the patients differ either with regard to the disease (acute exacerbation versus chronic stages, slow versus rapid decline of lung function), to co-morbidities (hypertension, viral infection, chronic aspiration of gastric content) or to other trigger factors such as age (age-related mitochondrial and peroxisomal dysfunction leading to oxidative stress), environmental exposures, smoking, and genetic factors [53]. Interestingly, differences between patients in our experiments were mainly observed for protein levels of PPAR-α (Fig. 1D), PPAR-γ (Fig. 1D), MMP-1 (Additional file: Fig. S1C) and catalase (Fig. 6C), whereas the protein levels of PPAR-β/δ (Fig. 1D), catalase activity (Fig. 6E), the level of intracellular and secreted collagen with and without TGF-β1 (Figs. 1B,  2B–D, Additional file: Fig. S1D) as well as the collagen-reducing effect of a combined treatment with PPAR-β/δ and PPAR-γ agonists (Fig. 3A–F) were less variable. This gives hope that the observed beneficial effect of PPAR-β/δ and PPAR-γ agonists is applicable to a broad spectrum of IPF patients. However, the strong heterogeneity of the target, namely the fibroblasts of IPF, but also of control patients, will limit the global use of any drug for IPF. Clinical trials discriminating between different subsets of patients may help to find the right drug in this regard.

We demonstrated that among the three PPARs, PPAR-β/δ might be a strong target for lung fibrosis resolution compared to PPAR-α (minor effect) and PPAR-γ (additive effect with PPAR-β/δ under these experimental conditions, Table 2). Focusing first on fibrosis pathways, we detected no differences between control and IPF fibroblasts with regard to the synthesis and release of collagen as well as gene expression and activity of MMP-1 (the dominant MMP, Fig. 4A) either when treated or untreated with TGF-β1, and PPAR-β and PPAR-γ agonists*.* However, MMPs differ between the diverse lung cell types such as alveolar epithelial type I and type II cells, alveolar macrophages and endothelial cells [54, 55]. In addition, MMP-1, -2, -3, -7, -13, -14, and -19, exhibit either anti- or pro-fibrotic [28] activities. MMP-2, as an example for the latter one, cleaves elastin which is deleterious for the lung. Interestingly, PPAR-β stimulation decreased the secretion of MMP-2 and increased the elastin level in human skin fibroblasts [56].

**Table 2:**
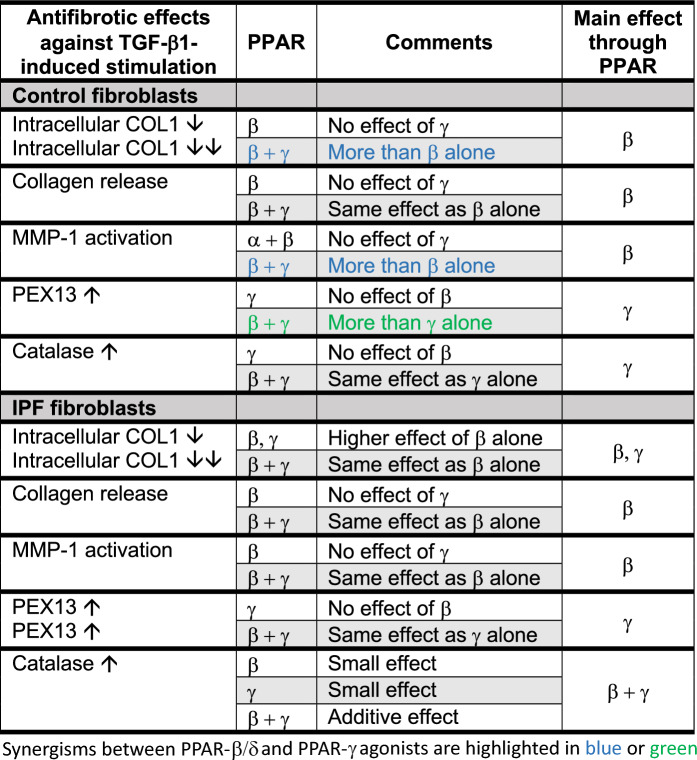
Summary of the respective PPARs responsible for the reversal of distinct TGF-β1-induced fibrotic alterations in human control and IPF fibroblasts

Next, we observed a TGF-β1-induced decrease in the peroxisomal biogenesis protein PEX13 which is reversed by stimulation of PPAR-γ. This was accompanied by changes in peroxisomal lipid metabolism, e.g. TGF-β1 increased the level of phosphatidylcholine in control, but decreased it in IPF fibroblasts with no additional effects of the PPAR drugs. The levels of AA, DHA and EPA were not significantly changed by TGF-β1, but increased strongly upon treatment with the PPAR-γ agonist. Metabolites from AA oxidation have been described to mediate inflammatory responses, and DHA is known to be anti-inflammatory [57, 58]. A balance between the fatty acids will essentially determine the direction of the drug interventions. The production of DHA was more than that of AA in control and IPF fibroblasts following PPAR-γ activation, whereas the activation of PPAR-β/δ increased levels of AA to a higher extent compared to DHA in control and IPF fibroblasts. However, the strong anti-fibrotic effects of PPAR-β/δ support the combined activation of both receptors during treatments. Thus, with regard to peroxisomes, PPAR-β/δ and PPAR-γ agonists increased the peroxisomal biogenesis protein PEX13, as well as peroxisome lipid metabolism, and the resulting metabolites may further activate PPARs, establishing a positive activation loop [59, 60].

Furthermore, the TGF-β1-induced decrease in the protein level and activity of catalase was reversed upon stimulation of PPAR-γ and PPAR-β/δ. Interestingly, in control fibroblasts the anti-fibrotic effect is mediated mainly via the maintenance of catalase protein through a reactive oxygen species (ROS)-dependent stimulation of PPAR-γ, because the effect is blocked by the specific catalase inhibitor AT in the combined treatment group by sustaining catalase levels. In IPF fibroblasts, the anti-fibrotic effect is mainly caused by a *combined* activation of PPAR-β/δ and PPAR-γ. The collagen-reducing effect is not inhibited by AT and thus ROS-independent. A decreased catalase level has been found in lung homogenates (and especially in the bronchial epithelium) of patients with IPF [61]. In acatalasemic mice, bleomycin induced a much higher invasion of pro-inflammatory cells together with increased levels of TGF-β1 and collagen and thus a higher degree of fibrosis [29], suggesting a beneficial role of high catalase levels in IPF disease progression. Interestingly, catalase (low affinity, high turnover) together with PRDX1 and PRDX5 (high affinity, low turnover), breakdown H_2_O_2_ generated by multiple pathways inside peroxisomes. While catalase is crucial for safeguarding the organelle at excessive H_2_O_2_, PRDX1 and PRDX5 function as a redox-regulator in cell signaling and H_2_O_2_ redox relay factor at low levels of H_2_O_2_, respectively [62]. In addition, catalase impedes ROS-induced inhibition of peroxisomal β-oxidation including the synthesis of the anti-inflammatory DHA [61]. With regard to PPARs, the catalase gene promotor region contains PPRE binding sites, e.g. for PPAR-γ (located at nucleotides − 1027 to − 1014; [63]) and an additional PPAR-γ binding site in humans only (located at nucleotides − 11,710 to − 11,698, [64]). Activation of PPAR-γ [23], but also of PPAR-β/δ (at the direct repeat 1 response element, [65]) increased catalase protein levels [65, 66]. We assume that the observed increase in catalase protein in our experiments by PPAR-β/δ and PPAR-γ was similarly due to an induction of the catalase promotor activity. The additive effect by the combined treatment with PPAR-β/δ and PPAR-γ ligands in IPF fibroblasts suggests an importance of the additional human-specific PPRE binding sites and demonstrates that human models are required to analyze the role of PPARs in fibrosis.

We would like to emphasize that in contrast to most of the previous publications we performed a *post-treatment* (to mimic the clinical situation) with a combination of PPAR-β/δ and PPAR-γ agonists to reverse the TGF-β1-induced fibrotic phenotype of IPF fibroblasts. It is well known that activated PPAR-γ alone is potentially anti-fibrotic [17–20]. With regard to PPAR-β/δ, to the best of our knowledge, only one review described an inhibition of the proliferation of normal human lung fibroblasts by its stimulation [26]. The question arises how an activation of PPAR-β/δ can support PPAR-γ or vice-versa. One possibility is that stimulation of one PPAR might increase the protein level of itself and of the other receptors. For example, agonists for PPAR-α and PPAR-β/δ, but not PPAR-γ, have been shown to increase the protein levels of PPAR-β/δ and PPAR-γ in osteoblasts [37]. Thus, especially PPAR-β/δ stimulation can end up in a positive activation loop as it increased its own as well as the PPAR-γ receptor [60]. This offers the possibility for a post-treatment schedule starting with the PPAR-β/δ agonist to increase PPAR-γ levels so that the later given PPAR-γ agonist can work more efficiently. Interestingly, after 48 h treatment with TGF-β1, we observed increases in the protein levels of PPAR-γ and PPAR-β/δ in control and IPF fibroblasts although with varying degrees (Fig. 2E, Table 1). This might explain why the *post-treatment* with PPAR-β/δ and PPAR-γ agonists is even more beneficial than *direct* treatment. Moreover, we demonstrated that the test compound STK 648389 (ZINC ID: 31,775,965), which has been suggested to be a dual PPAR-β/δ/PPAR-γ agonist by structure-based virtual screening [67], did not elicit anti-fibrotic effects (Additional file: Fig. S2). We hypothesized that the dual agonist (which is a single molecule) might be less specific for both receptors than the respective individual agonists and must be applied at a higher concentration which could induce more side effects in lung fibroblasts. Indeed, luciferase transactivation assays have shown EC50 values of 132 µM for PPAR-β/δ and 18 µM for PPAR-γ [67], and thus STK 648389 activated PPAR-γ only (see Fig. 3E showing no reduction of the extracellular collagen using 10 µM of the specific PPARγ agonist troglitazone).

In summary, combined activation of PPAR-β/δ and PPAR-γ exerts strong anti-fibrotic effects. Catalase, which is decreased during treatment with TGF-β1, is inverse proportionally involved in collagen production. Catalase protein level and activity can be increased by stimulation of PPAR-β/δ and PPAR-γ in control and IPF human lung fibroblasts. For IPF patients (to refer to the clinical situation), the most beneficial anti-fibrotic effects could possibly be achieved by a combined local treatment with PPAR-β/δ and PPAR-γ agonists via aerosol inhalation.

## Supplementary Information

Detailed description of the methods, 8 additional files.

## Data Availability

Raw data of the lipid analyses are available upon request to the corresponding author.

## Abbreviations

α-SMA: α-Smooth muscle actin
AA: Arachidonic acid
AT: 3-Amino-1,2,4-triazole
CAT: Catalase
COL1: Collagen type 1
DMEM: Dulbecco’s Modified Eagle Medium
DHA: Docosahexaenoic acid
ECM: Extracellular matrix
EPA: Eicosapentaenoic acid
GPX1/2: Glutathione peroxidase 1/2
HRP: Horse radish peroxidase
IPF: Idiopathic pulmonary fibrosis
LC–MS/MS: Liquid chromatography tandem mass spectrometry
MMP: Matrix metalloproteinase
MTBE: Methyl-*tert*-butyl ether
PC: Phosphatidylcholine
PE: Phosphatidylethanolamine
PEX: Peroxisomal biogenesis protein, peroxin
PPAR: Peroxisome proliferator-activated receptor
PRDX: Peroxiredoxin
PPRE: PPAR response element
ROS: Reactive oxygen species
RT-qPCR: Quantitative reverse transcription polymerase chain reaction
SM: Sphingomyelin
SPE: Solid phase extraction
TG: Triglycerides
TGF-β1: Transforming growth factor-beta 1
TGFBR1: Transforming growth factor-beta receptor 1
TNF-α: Tumor necrosis factor-α
