# Supplementary material for: It takes two peroxisome proliferator-activated receptors (PPAR-β/δ and PPAR-γ) to tango idiopathic pulmonary fibrosis
Source: Respir Res. 2024 Sep 23;25:345. doi: 10.1186/s12931-024-02935-7 (PMC11421181; doi:10.1186/s12931-024-02935-7)

**ONLINE DATA SUPPLEMENT**

**Methods**

Detailed description of the methods in the data supplement.

**Study approval**

Biospecimen collection was approved by the Ethics Committee of Justus Liebig University Giessen (Az58/15 and Az111/08, JLU), in accordance with laws binding research protocols in Germany. Primary human lung fibroblasts and tissues from organ donors (Additional file: Table S1) were collected in frame of the European IPF registry (eurIPFreg) and provided by the DZL/UGML Giessen Biobank (Deutsches Zentrum für Lungenforschung, Universities of Giessen and Marburg Lung Center). Lungs from the bleomycin-induced fibrosis mouse model were generous gifts from Dr. Michael Seimetz, Excellence Cluster Cardio-Pulmonary System, Giessen, Germany.

**Cell culture and drug treatment**

Frozen primary human lung fibroblasts from control and IPF patients were thawed and cultured in DMEM with 4.5 g/L D-glucose (Thermo Fisher Scientific, Paisley, UK), supplemented with 10% fetal bovine serum and penicillin (100 U/ml)/streptomycin (0.1 mg/ml, PAN Biotech, Aidenbach, Germany) at 37°C in a humidified atmosphere of 5% CO_2._ Catalase-deficient fibroblast cell lines were cultured in the same culture medium, but penicillin/streptomycin was replaced by puromycin (10 μg/ml, PAN Biotech, Aidenbach, Germany). For experiments, human lung fibroblasts were seeded at a density of 2 x 10^4^ cells/cm² into 12- or 24-well plates. At 80% confluency, cells were serum-starved for 3 h, stimulated with vehicle or rhTGF-β1 (R & D Systems, Cat. No. 240B) for 24 h (except for Figs. 2B, 2C, 2E, 3B), followed by the addition of the following drugs either alone or in combinations thereof for another 24 h: the PPAR-α agonist WY14643 (100 μM, Tocris Bioscience, Wiesbaden-Nordenstadt, Germany), the PPAR-β/δ agonist GW0742 (10 μM, Sigma-Aldrich, Taufkirchen, Germany), the PPAR-γ agonist rosiglitazone (10 μM, Tocris Bioscience, Wiesbaden-Nordenstadt, Germany) the PPAR-β/δ antagonist GSK0660 (10 nM, Tocris Bioscience, Wiesbaden-Nordenstadt, Germany), the PPAR-γ antagonist GW9662 (10 µM, Tocris Bioscience, Wiesbaden-Nordenstadt, Germany), the broad spectrum protease inhibitor 4-aminobenzoyl-Gly-Pro-D-Leu-D-Ala hydroxamic acid (20 µM, Sigma-Aldrich, Taufkirchen, Germany), the specific catalase inhibitor 3-amino-1,2,4-triazole (AT; 10 mM, Sigma-Aldrich, Taufkirchen, Germany), and the dual PPAR-β/δ and PPAR-γ agonist STK 648389 (25 and 50 µM; Vitas-ID; <https://vitasmlab.biz/>; Vitas-M Laboratory, delivered from Champaign, IL, USA; ZINC-ID 31775965).

**Generation of a stable catalase knockdown cell line**

Transduction with pGIPZ-shCatalase and pGIPZ-non-silencing control lentivirus vectors was done as described in an earlier publication [31]. Calcium phosphate was used to transfect the second generation packaging and envelope plasmids (psPAX2 and pCI-VSVG) together with lentiviral plasmids into the human embryonic kidney cell line, HEK293T. Cell supernatant containing the lentiviruses was collected over 2 days after transfection and further concentrated by ultracentrifugation at 52,000 rpm for 4 h in the Sorvall WX80+ ultracentrifuge (Thermo Fisher Scientific, Germany) using the AH-629 rotor. Virus titer was determined using fluorescence-based titration in human GBM line G55TL. To generate a catalase knockdown, the fibroblasts were transduced at a multiplicity of infection ratio of 50 with pGIPZ-sh*CAT* and pGIPZ-non-silencing lentivirus vectors (Dharmacon Reagents, Lafayette, USA) and afterwards selected with puromycin.

**Knockdown of catalase in control and IPF primary human lung fibroblasts using the siRNA technology**

Knockdown of catalase was done with CAT siRNA (Dharmacon Reagents, Lafayette, USA); negative control siRNA was from Qiagen, Hilden Germany. Control and IPF primary human lung fibroblasts were seeded at a density of 4 x 10^4^ cells/cm² (only for transfection experiments), Cells were transfected with 30 nmol siRNA using ScreenFectA transfection reagent (Incella, Eggenstein-Leopoldshafen, Germany) following the manufacturer’s instructions and were used for experiments 48 h later.

**Overexpression of catalase in control and IPF primary human lung fibroblasts** Transfection of fibroblasts with a catalase overexpression plasmid (pGL 4.14-Catalase) and promoter reporter plasmids *COL1A2*-luc and *PPRE*-luc followed the same routine as already described [13, 32]. Data from pRL-SV40 Vector (Promega, Mannheim, Germany) was used to normalize results for the luciferase reporter plasmid. One volume of Cell Lysis Reagent (Promega, Mannheim, Germany) was diluted in 4 volumes of sterile water and added to each well, swirled, scraped with a rubber policeman, and lysates were collected for measurements of the Renilla and firefly luciferase activities.

**BrdU cell proliferation assay**

Control and IPF primary human lung fibroblasts were seeded at a density of 6 x 10^4^ cells/cm² into 96-well plates and treated with vehicle or TGF-β1 (5 ng/ml) for 24 h. BrdU was added later to the wells. Cell proliferation was determined following the manufacturer’s instructions (BrdU Cell Proliferation Kit, 2750, Millipore, Schwalbach, Germany); color development was measured at 450 nm.

**Human TGF- β1 immunoassay**

Control and IPF primary human lung fibroblasts were seeded at a density of 4 x 10^4^ cells/cm² in 24-well plates. Cells were serum-starved for 24 h and cell culture media were collected for centrifugation to remove debris. Supernatants were separated from the pellets and used for the assay following instructions provided by the manufacturer (DB100C, Quantikine^TM^ ELISA, R&D Systems, Minneapolis, USA).

**Protein isolation and Western blotting**

Drug-treated and/or siRNA-transfected control and IPF primary human lung fibroblasts were washed with PBS (PAA laboratories GmbH, Pasching, Austria) and 100 μl of cell lysis buffer supplemented with protease inhibitor mix was added to the 12-well plates. Lysates were collected for separation in 10% SDS-PAGE gel at reducing conditions, blotted on polyvinylidene fluoride (PVDF) membranes (Millipore, Schwalbach, Germany) and detections were made following incubation with primary and secondary antibodies against proteins of interest. Primary antibodies were used are as follows: rabbit anti-PEX13 IgG (1:1000, generous gift from Denis Crane, Brisbane, Australia), rabbit anti-PPAR-α IgG (1:100; sc1985, Santa Cruz, Heidelberg, Germany), rabbit anti-PPAR-β/δ IgG (1:1000; R22951, Abiocode, Agoura Hills, USA), rabbit anti-PPAR-γ IgG (1:100, sc7196, Santa Cruz, Heidelberg, Germany), rabbit anti-catalase IgG (1:200, A21260-1AP, Proteintech, Rosemont, USA), rabbit anti-TGFBR1 IgG (1:500, ab121024, Abcam, Cambridge, UK), rabbit anti-MMP-1 IgG (1:200, 10371-2-AP, Proteintech, Rosemont, USA), rabbit anti-COL1 IgG (1:200, 14695-1-AP, Proteintech, Rosemont, USA), mouse anti-GAPDH IgG (1:10,000, 5G4, HyTest, Turku, Finland), mouse anti-β-actin IgG (1:8000, A5316, Sigma-Aldrich, Taufkirchen, Germany), mouse anti-α-tubulin IgG (1:5000, T5168, Sigma, Darmstadt, Germany), and mouse anti α-SMA IgG (1:2000, A2547, Sigma-Aldrich, Taufkirchen, Germany). PVDF membranes were incubated with HRP-linked secondary antibodies, that is, rabbit anti-mouse IgG (1:10,000, A9044, Sigma-Aldrich, Taufkirchen, Germany) and goat anti-rabbit IgG (1:7000, A0545, Sigma-Aldrich, Taufkirchen, Germany), for 1 h at room temperature. Clarity ^TM^ western ECL (Bio-Rad Laboratories, Feldkirchen, Germany) was used to visualize bands on membranes. Semi-quantitative analyses of signal intensities were done with ImageJ.

**Immunofluorescence staining**

Paraffin-embedded lung tissue blocks from control and IPF patients were cut into sections of 2-3 μm thickness and placed in an oven set at 50°C overnight. The sections were deparaffinized with xylene, rehydrated in decreasing gradients of ethanol, and digested with trypsin. Unspecific binding sites were blocked with 4% PBSA for 2 h at room temperature, followed by an incubation overnight at 4°C with the following primary antibodies: rabbit anti-COL1 IgG (1:50; 14695-1-AP, Proteintech, Rosemont, USA), mouse anti-α-SMA IgG (1:2000; A2547, Sigma-Aldrich, Taufkirchen, Germany), rabbit anti-catalase IgG (1:50, A21260-1-AP, Proteintech, Rosemont, USA) and mouse anti-glutathione peroxidase 1/2 IgG (1:200; sc-74498; Santa Cruz, Heidelberg, Germany). The next day, tissue sections were washed and incubated with secondary antibodies for 1 h at room temperature using goat anti-mouse IgG coupled with Alexa Fluor 555 (1:1000, A-21429, Molecular Probes/Invitrogen, Waltham, USA) or donkey anti-rabbit IgG coupled with Alexa Fluor 488 (1:1000, Invitrogen A-21206, Molecular Probes/Invitrogen, Waltham, USA). Nuclei were counterstained with DAPI.

**Isolation of total RNA and RT-qPCR**

Total RNA was isolated from cultured control and IPF primary human lung fibroblasts using RNAzol (R4533, Sigma-Aldrich, Taufkirchen, Germany) and cDNA was synthesized using the high-capacity cDNA reverse transcription kit (4368814, Thermo Fisher Scientific, Germany). The mRNA levels of the respective genes were analyzed by RT-qPCR using the iQ^TM^ SYBR Green Supermix (4368814, Bio-Rad Laboratories, Feldkirchen, Germany) and normalized to *HPRT1* as reference gene. The sequences of primers together with their efficiency coefficients are given in Additional file: Table S2.

**Sircol collagen assay**

Control and IPF primary human lung fibroblasts were cultured in 24-well plates and the culture media were collected for Sircol collagen assay following the manufacturer’s (Biocolor, Newtownabbey, UK) instructions. Color development was detected at a wavelength of 570 nm in a TriStar LB 941 Multimode Microplate Reader. Collagen concentrations of samples were extrapolated from a line of best fit obtained from standards.

**Catalase and hydrogen peroxide assays**

Cells were treated, washed and homogenized in PBS. Catalase activity was determined using an assay kit (KA1626, Abnova, Heidelberg, Germany) following instructions given by the manufacturer. Hydrogen peroxide released by the cells was measured using the culture media with an assay kit (CAY600050, Cayman Chemical, Ann Arbor, USA), following the instructions provided.

**Materials for lipid analyses and targeted quantification of fatty acids**

Arachidonic acid (AA), docosahexaenoic acid (DHA), eicosapentaenoic acid (EPA), and deuterated internal standard arachidonic acid-d11 (AA-d11) were purchased from Cayman Chemical Company (distributor: Biomol GmbH, Hamburg, Germany). Strata-X polymeric reversed-phase SPE cartridges (33 μm, 100 mg/3 ml) were procured from Phenomenex (Torrance, USA). MS grade solvents (water, acetonitrile, methanol, 2-propanol) and formic acid were obtained from Honeywell Riedel-de Haën (Seelze, Germany), and methyl-*tert*-butyl-ether (MTBE) and ammonium formate from Sigma-Aldrich (Taufkirchen, Germany). Control and IPF primary human lung fibroblasts were seeded and cultured in 6-well plates for 24 h. At confluency, cells were serum-starved for 3 h and treated with TGF-β1 (5 ng/ml) for 24 h, followed by the addition of PPAR ligands for another 24 h. Afterwards, cell supernatants were collected and spiked with deuterated internal standard and fatty acids extracted with a solid phase extraction (SPE) method as described previously [32]. SPE cartridges were equilibrated with methanol, followed by water; then the samples were loaded and washed with 10% methanol and eluted with 100% methanol. The methanol eluates were dried down using a nitrogen gas stream and reconstituted with 25% acetonitrile. The fatty acid species were identified and quantified using liquid chromatography (Dinoex Ultimate 3000 UHPLC, Thermo Fisher Scientific, Germany) interfaced with an orbital trapping mass spectrometer (Q Exactive, Thermo Fisher Scientific, Germany). In brief, 10 µl of the reconstituted extract was injected and separated using a reversed-phase Kinetex C18 2.6-μm column (100 × 2.1 mm, 100 Å) with a flow rate of 500 μl/min. The binary solvent system consisting of mobile phase A (100% water with 0.1% formic acid) and mobile phase B (100% acetonitrile with 0.1% formic acid). The MS data was acquired in negative-ion mode with a linear gradient of over 10 minutes, and the targeted quantitative analysis was performed as described previously [32].

**Untargeted lipidomics**

The cell pellets were re-suspended in ice-cold water (MS grade) and protein concentrations measured by Bradford assay. Lipids were extracted from cell lysates using a biphasic MTBE extraction [33]. Briefly, an equal protein amount of cell lysates were transferred to a glass vial and MS grade ice-cold methanol, MTBE and water were added to a final ratio of 1:3:1 (v/v/v). Samples were vortexed for 1 h and then centrifuged for 15 min at 13,500 x *g* at 4°C. After centrifugation, the upper layer was collected and dried down using a speed vac at room temperature. The dried lipid extracts were reconstituted in 100 μl of acetonitrile/water/2-propanol (65:5:30, v/v/v) and analyzed by using liquid chromatography tandem mass spectrometry (LC-MS/MS) [34]. Briefly, 10 μl of lipid extract was injected into Ultimate 3000 UHPLC coupled to a hybrid quadrupole orbital trapping mass spectrometer (Q Exactive, Thermo Fisher Scientific, Bremen, Germany). Separation of lipids was achieved using a reversed-phase HSS T3 1.8 μm column (100 × 2.1 mm) with a flow rate of 250 μl/min maintained at 50°C. The binary solvent system consisted of mobile phase A (water: acetonitrile, 40:60 v/v with 10 mM ammonium formate and 0.1% formic acid) and mobile phase B (2-propanol: acetonitrile, 90:10 v/v with 10 mM ammonium formate and 0.1% formic acid). All data were acquired in both positive- and negative-ionization modes (biological triplicates) with a linear gradient over a run time of 28 min using data-dependent MS2 acquisition mode. Lipid Search 4.2.21 software was used for lipid identification and relative-quantification with parameters as described previously [34].

**Statistics**

Statistical analysis was done using GraphPad Prism 6 software. All data were obtained from at least three individual experiments and values were expressed as means +/- SEM. For comparison of the outcome between two groups, the F-test was first applied to compare their variances followed by Mann-Whitney U test (unequal variances) or unpaired Student’s *t*-test (equal variances) to calculate *p*-values. For multiple comparisons, either one-way or two-way ANOVA were used with a post-hoc Tukey´s multiple comparisons test to calculate *p*-values between individual groups. All *p*-values were set for **p*<0.05, ***p*<0.01, ****p*<0.001 and *****p*<0.0001.

**Supplemental Figure S1**


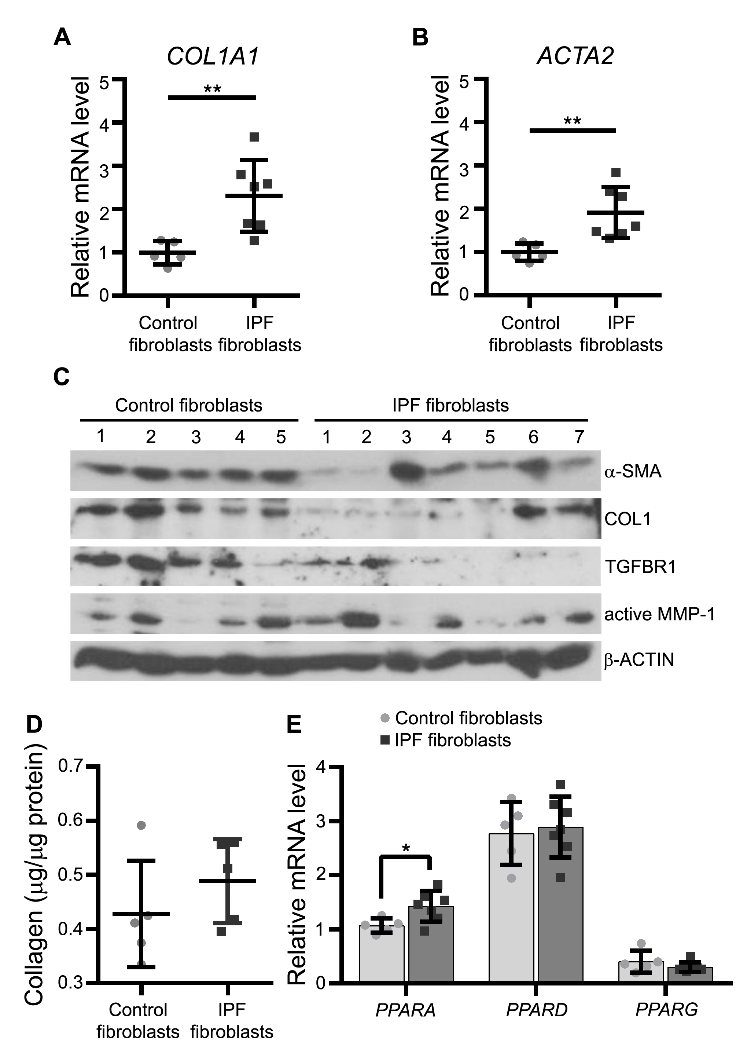


**Fig. S1** Variable protein levels of α-SMA, COL1, TGF-β1 receptor 1 (TGFBR1), and active MMP-1 as well as in the release of collagen by fibroblasts from control and IPF patients. **A-E** Cell lysates were used to determine the mRNA levels of *COL1A1* (**A**), *ACTA2* (**B**), *PPARA, PPARD and PPARG* (**E**) by RT-qPCR using *HPRT1* as reference gene, and the protein levels of α-SMA, COL1, active MMP1 and TGFBR1 (**C**) for Western blot analysis from fibroblasts of 5 control and 7 IPF patients using β-Actin (β-ACTIN) as reference protein. **D** The release of collagen into the culture media was measured using Sircol assay. Data represent 5 control and 5 IPF patients across six independent fibroblast cultures and are given as µg collagen/µg cell protein.

**Supplemental** **Figure S2**


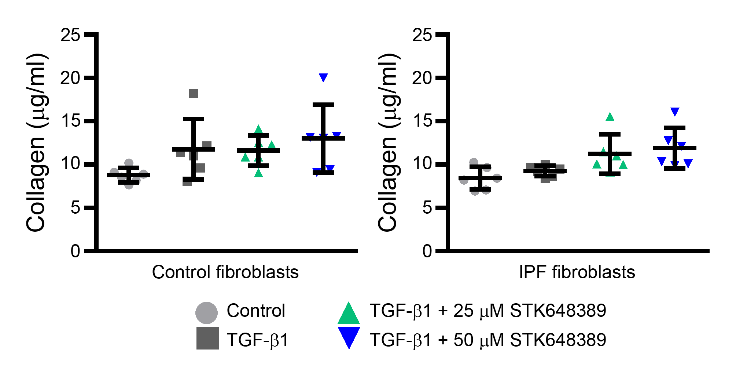


**Fig. S2** The dual PPAR-β/δ and PPAR-γ agonist STK 648389 did not decrease extracellular collagen produced by TGF-β1-stimulated control and IPF fibroblasts. Cells were serum-starved for 3 h, stimulated with vehicle (Control) or TGF-β1 (5 ng/ml) for 24 h, followed by the addition of STK 648389 (25 and 50 μM) for another 24 h. Culture media were collected and extracellular collagen was analyzed using Sircol assay.

**Supplemental Figure S3**


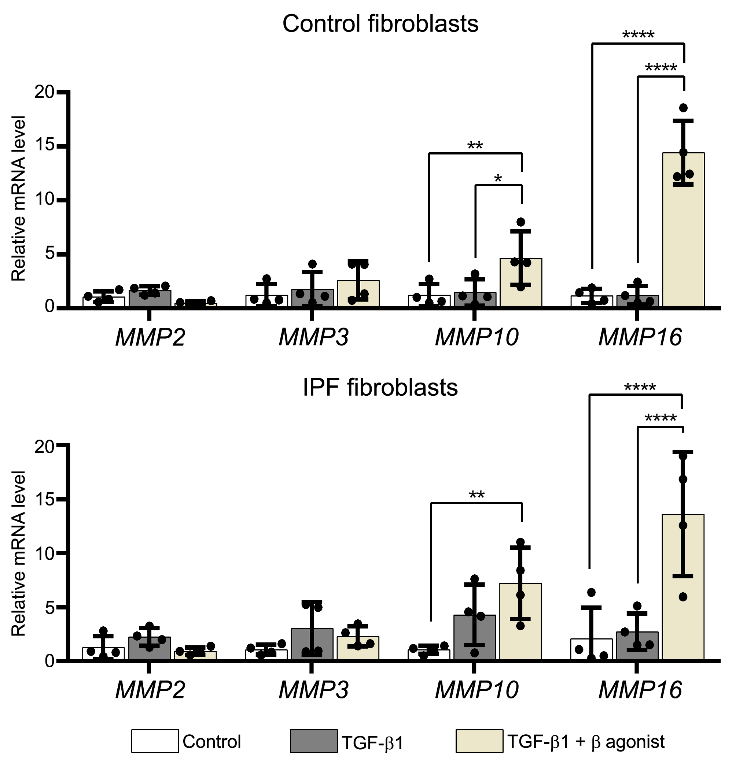


**Fig. S3** Ligand activation of PPAR-β/δ strongly increased the mRNA levels of *MMP10* and *MMP16* in TGF-β1-treated control and IPF fibroblasts. Cells were serum-starved for 3 h, stimulated with vehicle (Control) or TGF-β1 (5 ng/ml) for 24 h, followed by the addition of the PPAR-β/δ agonist GW0742 (10 μM, β) or vehicle for another 24 h. Cells were collected and the mRNA levels were measured by RT-qPCR with *HPRT1* as reference gene.

**Supplemental Figure S4**


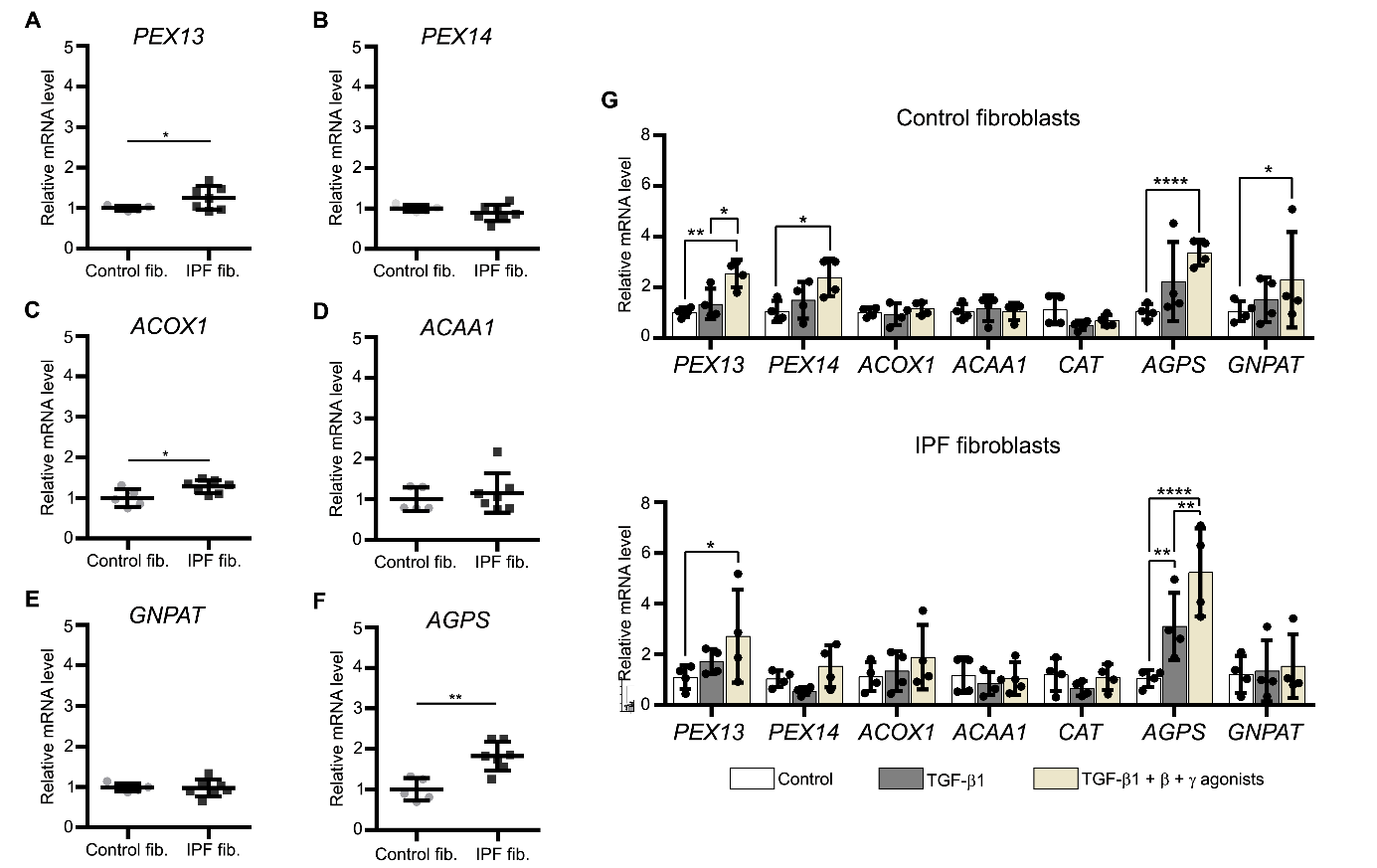


**Fig. S4** Combined treatment with PPAR-β/δ and PPAR-γ agonists which were added 24 h after TGF-β1, stimulated peroxisome biogenesis in control and IPF fibroblasts. **A**-**F** Comparative expression profile of selected peroxisomal genes was done by RT-qPCR with *HPRT1* as reference gene. **G** Ligand activation of PPAR-β/δ in combination with PPAR-γ increased the mRNA level of some peroxisomal genes in TGF-β1-treated control and IPF fibroblasts. Cells were serum-starved for 3 h, stimulated with vehicle (Control) or TGF-β1 (5 ng/ml) for 24 h, followed by the addition of agonists of PPAR-β/δ GW0742 (10 μM, β) and PPAR-γ rosiglitazone (10 μM, γ) or vehicle for another 24 h. The mRNA levels were analyzed by RT-qPCR with *HPRT1* as reference gene.

**Supplemental Figure S5**


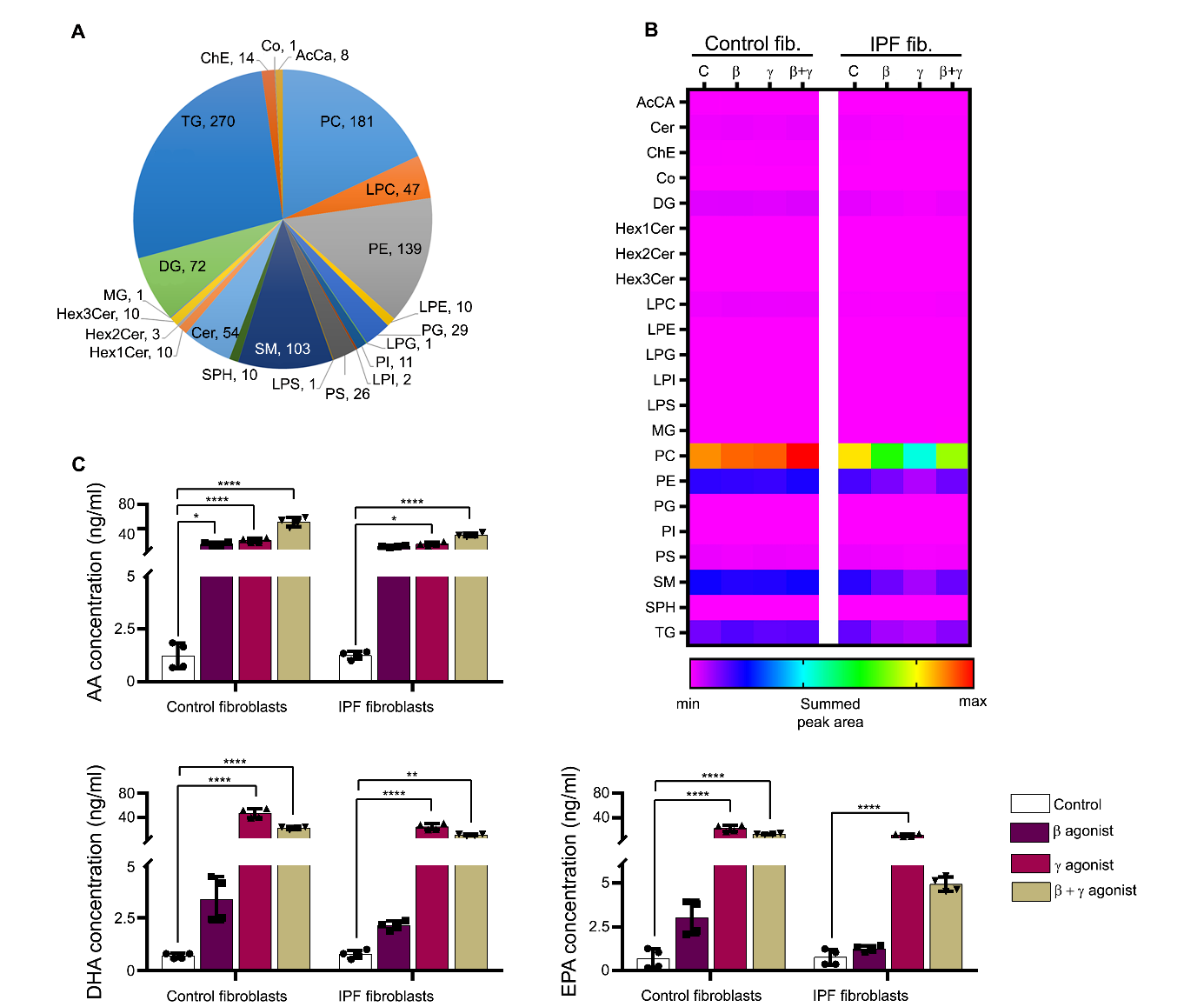


**Fig. S5** Combined treatment with PPAR-β/δ and PPAR-γ agonists which were added 24 h after TGF-β1, stimulated peroxisome lipid metabolism in control and IPF fibroblasts. **A** Summary of total lipid species identified in fibroblasts using liquid chromatography-tandem spectrometry (LC-MS/MS). **B** Heatmap of the lipidomic profile fibroblasts from control and IPF patients. Cells were serum-starved for 3 h and then treated for 48 h with vehicle (Control), the PPAR-β/δ agonist GW0742 (10 μM, β) and the PPAR-γ agonist rosiglitazone (10 μM, γ) either alone or combined. Cells were collected in PBS for lipid analysis using LC-MS/MS. **C** Fibroblasts from control and IPF patients were serum-starved for 3 h, followed by the addition of vehicle (Control), the PPAR-β/δ agonist GW0742 (β, 10 μM) and the PPAR-γ agonist rosiglitazone (10 μM, γ) either alone or combined for 48 h. The releases of AA, DHA, and EPA were analyzed in the culture media by LC-MS/MS.

Abbreviations in **B**: AcCa - Acyl Carnitines, Cer - Ceramide, ChE - Cholesterol esters / Cholesterol, Co - Coenzyme, DG - Diglyceride, Hex1Cer, Hex2Cer and Hex3Cer - Hexosylceramides, LPC - Lysophosphatidylcholine, LPE - Lysophosphatidylethanolamine, LPG -Lysophosphatidylglycerol, LPI - Lysophosphatidylinositol, LPS - Lysophosphatidylserine, MG - Monoglyceride, PC - Phosphatidylcholine, PE -Phosphatidylethanolamine, PG - Phosphatidylglycerol, PI - Phosphatidylinositol, PS - Phosphatidylserine, SM - Sphingomyelin, SPH - Sphingosine, TG - Triglyceride.

**Supplemental Figure S6**

**
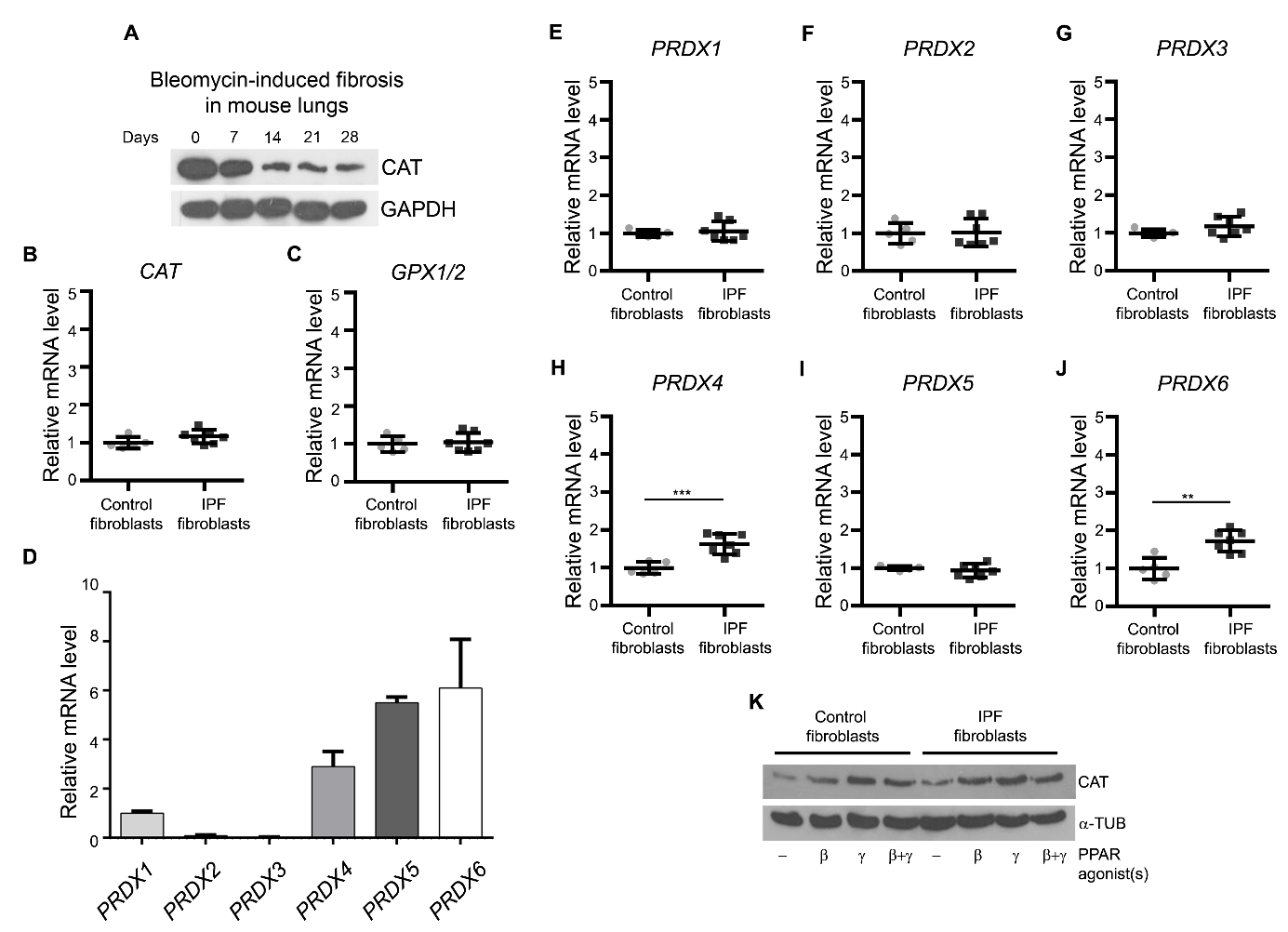
**

**Fig. S6** **A** Bleomycin administration decreased catalase protein levels in mouse lungs. **B**, **C** Comparison of the mRNA levels of *CAT* and *GPX1/2* genes between lung fibroblasts from control and IPF patients. Analysis of the mRNA levels was done by RT-qPCR with *HPRT1* as reference gene. **D**-**J** Comparative expression profile of each of the six *PRDX*s, the peroxiredoxins (PRDXs) of *PRDX1, PRDX2, PRDX3, PRDX4, PRDX5* and *PRDX6* of control fibroblasts (**D**) and between control and IPF fibroblasts (**F**-**J**). Analysis of the mRNA levels was done by RT-qPCR with *HPRT1* as reference gene. **K** Activation of PPAR-β/δ in combination with PPAR-γ increased catalase protein levels in control and IPF fibroblasts.

**Supplemental Figure S7**


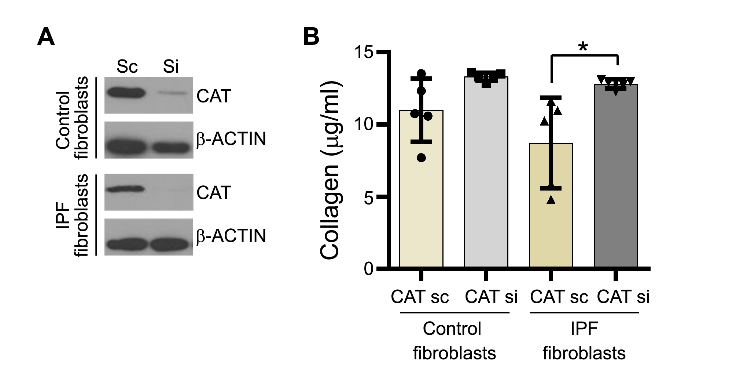


**Fig. S7** Knockdown of catalase using the siRNA technology induced the release of collagen by control and IPF fibroblasts. **A**, **B** Cells were transfected with scrambled (sc) and catalase (si) siRNAs for 48 h. Cell lysates were collected for Western blot analysis of catalase protein levels (CAT, **A**) with β-Actin (β-ACTIN) as reference protein and culture media for the analysis of extracellular collagen by Sircol assay (**B**).

**Supplementary Table S1.** Overview of the patient data**.**


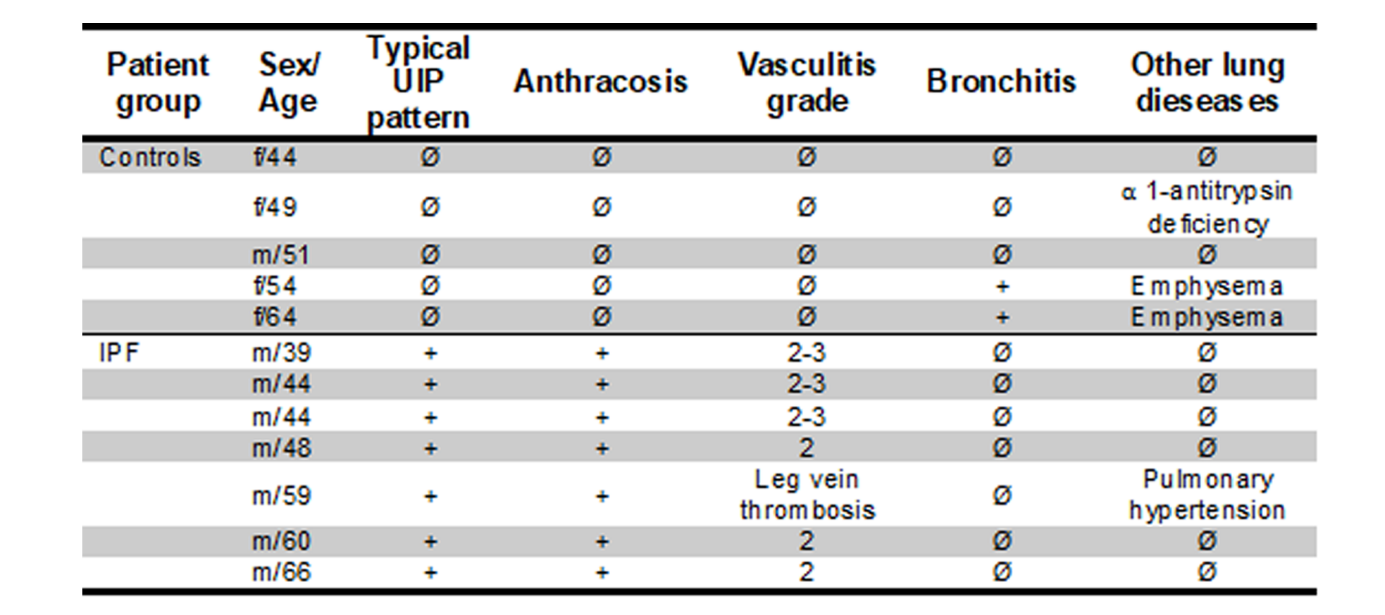


**Supplementary Table S2.** Primer sequences and efficiencies.


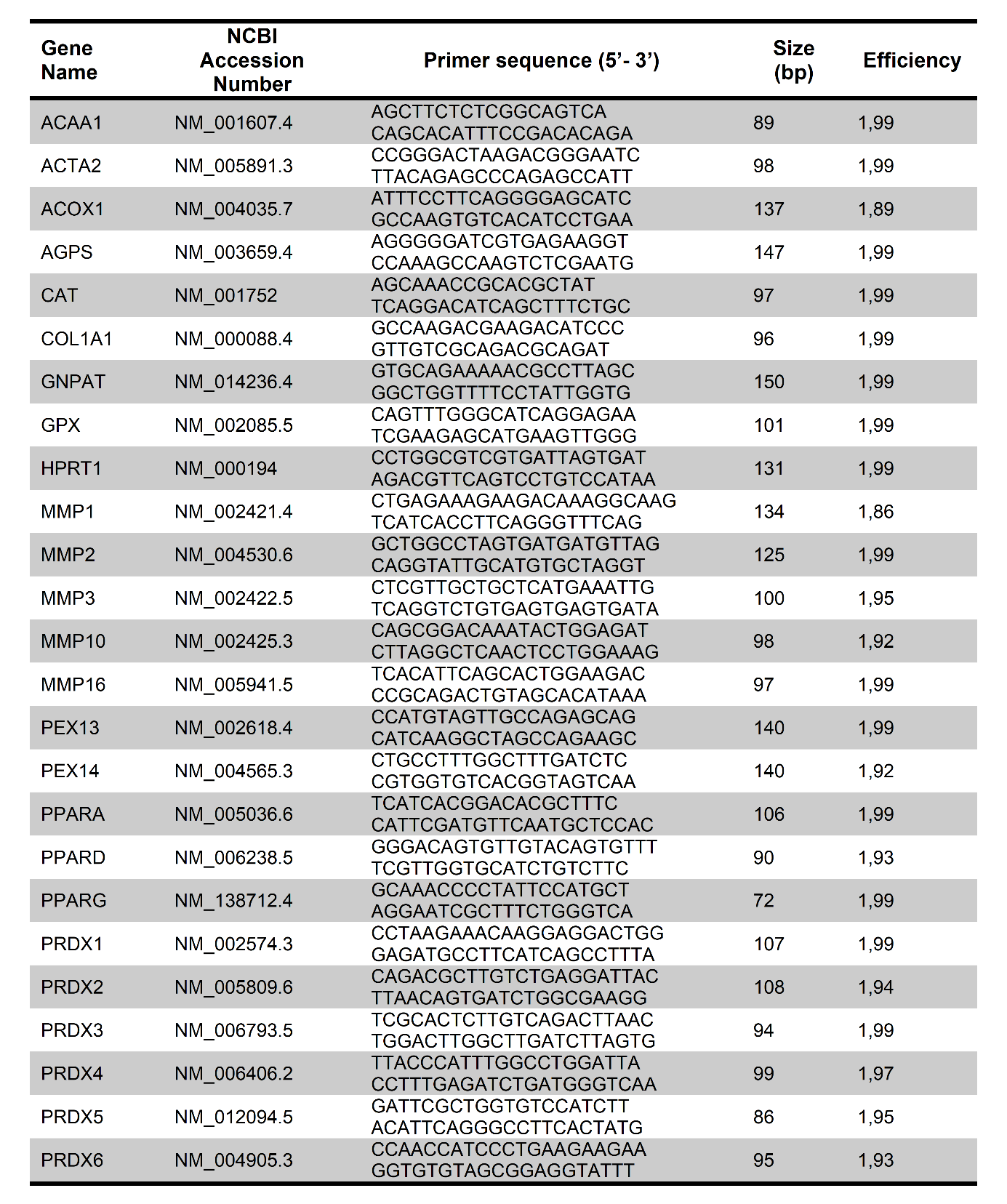

Supplement: Supplementary file 1 — Detailed description of the methods, 8 additional files. [file 12931_2024_2935_MOESM1_ESM.docx]
